# In Situ Proliferating Peptide Nanoparticle Augments Multi‐Target Intervention of Secondary Brain Damage Following Subarachnoid Hemorrhage

**DOI:** 10.1002/advs.202417456

**Published:** 2025-05-30

**Authors:** Yibin Zhang, Peisen Yao, Fuxiang Chen, Shufa Zheng, Xuegang Niu, Haojie Wang, Yuanxiang Lin, Bin Gao, Dezhi Kang

**Affiliations:** ^1^ Department of Neurosurgery Neurosurgery Research Institute The First Affiliated Hospital Fujian Medical University Fuzhou Fujian 350005 China; ^2^ Department of Neurosurgery National Regional Medical Center Binhai Campus of the First Affiliated Hospital Fujian Medical University Fuzhou 350212 China; ^3^ Fujian Provincial Institutes of Brain Disorders and Brain Sciences The First Affiliated Hospital Fujian Medical University Fuzhou Fujian 350005 China

**Keywords:** MMP‐2 responsiveness, multi‐target of combination, peptide nanoparticle, secondary brain injury, subarachnoid hemorrhage

## Abstract

Subarachnoid hemorrhage (SAH), a lethal stroke subtype, involves complex pathological cascades triggered by neuro‐glial units for persistent neuroinflammation, oxidative damage and programmed neuronal cell death. Single‐target and traditional multi‐target therapies, derived from individual drugs, show limited efficacy in addressing these interconnected events, due to spatiotemporal heterogeneity of action in single‐target components. This highlights the urgent need for not only new therapeutic targets, but advanced multi‐target drugs. Herein, we identify elevated cell‐free DNA (cfDNA), a key neuroinflammatory driver, as correlated with SAH severity and poor prognosis, suggesting its therapeutic potential. Furthermore, a novel “in situ proliferation” strategy is proposed and a flexible multi‐target peptide nanoparticle is developed through co‐assembling matrix metalloproteinase‐2 responsive cationic peptide and glutathione peroxidase‐mimicking peptide (GPXP). Upon reaching injury lesions, this system splits into two individual drugs: cationic peptide scavenges pathological cfDNA and inhibits microglia‐mediated neuroinflammation, while GPXP protects neurons against oxidative damage and neuronal apoptosis/ferroptosis. Consequently, this strategy proves superior therapeutic effects on reducing secondary brain injury and promoting neurofunctional recovery in SAH mice. These findings not only highlight the essential role of cfDNA in SAH but offer a flexible resolution to advance multi‐target combinational therapy.

## Graphical abstract

This work designs an in situ proliferating peptide nanoparticle as a flexible resolution that modulates the neuro‐glia unit to enable enhanced multi‐target combinational therapy for secondary brain damage following subarachnoid hemorrhage.

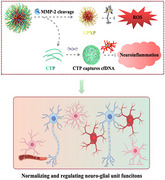

## 1 Introduction

Aneurysmal subarachnoid hemorrhage (SAH) represents only 5–10% of all stroke subtypes, but it has posed a severe threat to global public health with an estimated worldwide prevalence of ≈8.09 million cases.^[^ ^1^, ^2^, ^3^ ^]^ Even worse, SAH has brought the highest disability and mortality rates among all strokes.^[^ ^4^ ^]^ The reported mortality rate ranges from 25–50% and ≈50% of the survivors suffer from irreversible neurological impairments, i.e., long‐term or even permanent coma, neurological dysfunction, and disability.^[^ ^1^, ^4^, ^5^ ^]^ As a consequence, they still need long‐term rehabilitation, leading to a substantial economic burden on families and society. Furthermore, SAH is more prevalent among young adults compared to other types of strokes, which causes a significant reduction in their productivity and income.^[^ ^5^ ^]^ Despite advancements in approaches for the clinical diagnosis and treatment of SAH, the overall prognosis of SAH patients has not significantly improved.^[^ ^6^ ^]^ Therefore, it is imperative to investigate the pathophysiological mechanisms of brain injury after SAH and develop advanced therapeutic strategies to improve the prognosis of SAH patients.

Following SAH, the neuro‐glial unit is activated to trigger a series of pathophysiological changes, i.e., overproduction of reactive oxygen species (ROS), the elevated levels of inflammatory mediators, cascading oxidative damage, and programmed cell death of neurons, thereby contributing to a poor prognosis.^[^ ^1^, ^4^, ^7^, ^8^, ^9^ ^]^ The complex pathophysiology features represent a significant contributing factor to secondary brain injury (SBI), and they bring a large difficulty in developing pharmacological treatments.^[^ ^1^, ^6^, ^10^, ^11^ ^]^ Additionally, effective pharmacotherapies for SBI remain limited in clinical practice.^[^ ^1^, ^5^, ^6^, ^7^ ^]^ Consequently, this further highlights the imperative necessity for novel therapeutic targets and versatile multi‐target drugs capable of resolving the neuro‐glial unit dysfunction.

**Figure 1:**
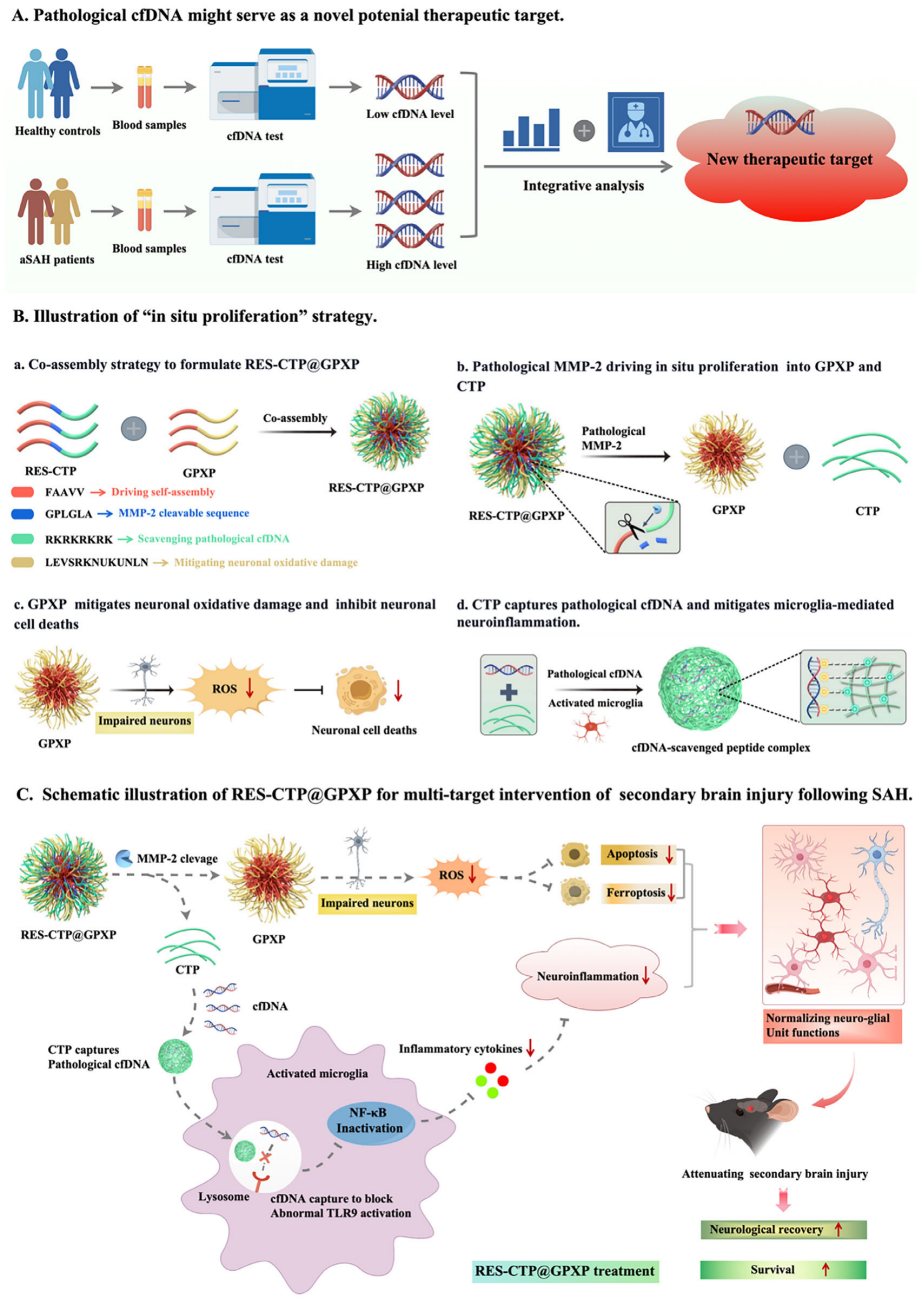
Schematic illustration of RES‐CTP@GPXP as a flexible multi‐targeted therapeutic for alleviating SAH‐induced SBI through in situ proliferation. A) Association of cfDNA levels with the severity of SBI in aSAH patients indicating that cfDNA might serve as a novel potential therapeutic target. B) Illustration of “in situ proliferation” strategy. C) RES‐CTP@GPXP system responds to the elevated MMP‐2 levels after SAH and then splits into the individual bioactive components of GPXP and CTP. CTP scavenges pathological cfDNA and inhibits microglia‐mediated neuroinflammation, while GPXP inhibits neuronal oxidative damage and neuronal cell death. The two components act synergistically to normalize the dysfunctions of the neuro‐glial unit, thereby achieving enhanced multi‐target therapy to reduce SBI following SAH.

Recent studies have underscored the critical involvement of cell‐free DNA (cfDNA) in the pathogenesis of various severe inflammatory diseases.^[^ ^12^, ^13^, ^14^, ^15^, ^16^ ^]^ Generally, the dying cells release cfDNA to bind Toll‐like receptor 9 (TLR9) in the subcellular lysosome of innate immune cells and then activate NF‐κB signaling pathway to trigger inflammatory responses.^[^ ^17^, ^20^ ^]^ In this work, we find that the elevated cfDNA levels are associated with diseaseseverity and poor 3‐month outcomes in patients with aneurysmal SAH (**Figure** **1**A). This discovery offers the possibility that pathological cfDNA may serve as a novel therapeutic target for mitigating neuroinflammation in SBI.

CfDNA‐scavenging has emerged as a promising approach to reduce pathological cfDNA levels and alleviate inflammatory diseases, where cfDNA‐scavenging materials are commonly designed as cationic types, i.e., cationic polymers and nanoparticles,^[^ ^12^, ^13^, ^21^, ^22^ ^]^ to electrostatically capture cfDNA and hinder its further recognition by TLR9.^[^ ^12^, ^13^, ^14^, ^15^, ^17^, ^23^, ^24^ ^]^ After establishing a positive correlation between cfDNA and SBI in patients, we intend to design a cfDNA‐scavenger specifically for alleviating microglia‐mediated neuroinflammation following aneurysmal subarachnoid hemorrhage (aSAH). Considering that oxidative damage and neuronal death also belong to the essential pathological factors of SBI progression, we try to develop a multi‐target therapeutic strategy to augment the therapeutic effect via overcoming a series of pathophysiological events induced by the neuro‐glial unit. However, one drug commonly modulates a single cell type or therapeutic target, which makes it difficult to simultaneously intervene in both the neuron and microglial cells, as well as the multiple pathophysiological events they jointly cause. Moreover, the conventional strategy for developing a multi‐target drug is mainly based on the simple integration of single‐target drugs, whose efficacy is still limited due to the spatiotemporal heterogeneity of action in individual single‐target components. This represents a huge technical challenge: how to rationally design this multi‐target drug to resolve the above question?

Benefiting from the designability, biological functions, self‐assembly property, and biocompatibility, peptides offer unique advantages in developing this multi‐target drug. To this end, we proposed an innovative “in situ proliferation” strategy, and further constructed a flexible multi‐target peptide nanoparticle via the supramolecular co‐assembly of matrix metalloproteinase‐2 (MMP‐2) responsive cationic peptide (RES‐CTP) and glutathione peroxidase‐mimicking peptide (GPXP) (Figure 1B). Their design principle was as follows: 1). the RES‐CTP was designed with a hydrophobic sequence to drive self‐assembly, an MMP‐2 cleavable sequence to respond to the elevated MMP‐2 level in lesions, and a cationic sequence to scavenge pathological cfDNA; 2). GPXP was designed with a hydrophobic sequence to drive self‐assembly, a selenium‐containing glutathione peroxidase‐mimicking sequence to alleviate oxidative damage, and neuronal cells undergoing apoptosis‐targeting sequence in hemorrhagic stroke. The co‐assembled system, termed RES‐CTP@GPXP, could reach brain lesions via intranasal administration and was responsive to the elevated levels of MMP‐2 for in situ proliferation into the two drugs (individual cationic peptide and GPXP), where the cationic peptide (CTP) scavenged pathological cfDNA and attenuated microglia‐mediated neuroinflammation. GPXP preferentially targeted neurons to inhibit oxidative damage and neuronal apoptosis/ferroptosis (Figure 1C). This in situ proliferating strategy was beneficial in maximizing the therapeutic potential of individual CTP and GPXP components, attributed to their independent modulation of different cell types and therapeutic targets.

Consequently, this in situ proliferating peptide nanoparticle was demonstrated to effectively disrupt the pathological feedback loop of the neuro‐glial unit via simultaneously intervening in neuron/microglial cells, as well as their jointly caused ROS overproduction, neuroinflammation, oxidative damage, and neuronal death. Its therapeutic effects in reducing SBI and promoting neurofunctional recovery were superior to single‐target therapy and simply‐combining dual‐target therapy in experimental SAH mice. This present work not only highlighted the essential role of cfDNA in SAH but offered an in‐situ proliferation concept for the design principle of multi‐target combinational therapy.

## 2 Results and Discussion

### 2.1 CfDNA Level was Associated with Brain Injury and Prognosis in Patients with aSAH

cfDNA, recognized as a damage‐associated molecular pattern (DAMP), can actively drive inflammatory reactions by activating resident immune cells.^[^ ^17^, ^25^ ^]^ Recent studies have confirmed cfDNA's diagnostic utility and suggested its potential as a therapeutic target to mitigate inflammation in multiple severe diseases associated with inflammation.^[^ ^12^, ^15^, ^17^, ^21^, ^23^ ^]^

**Figure 2:**
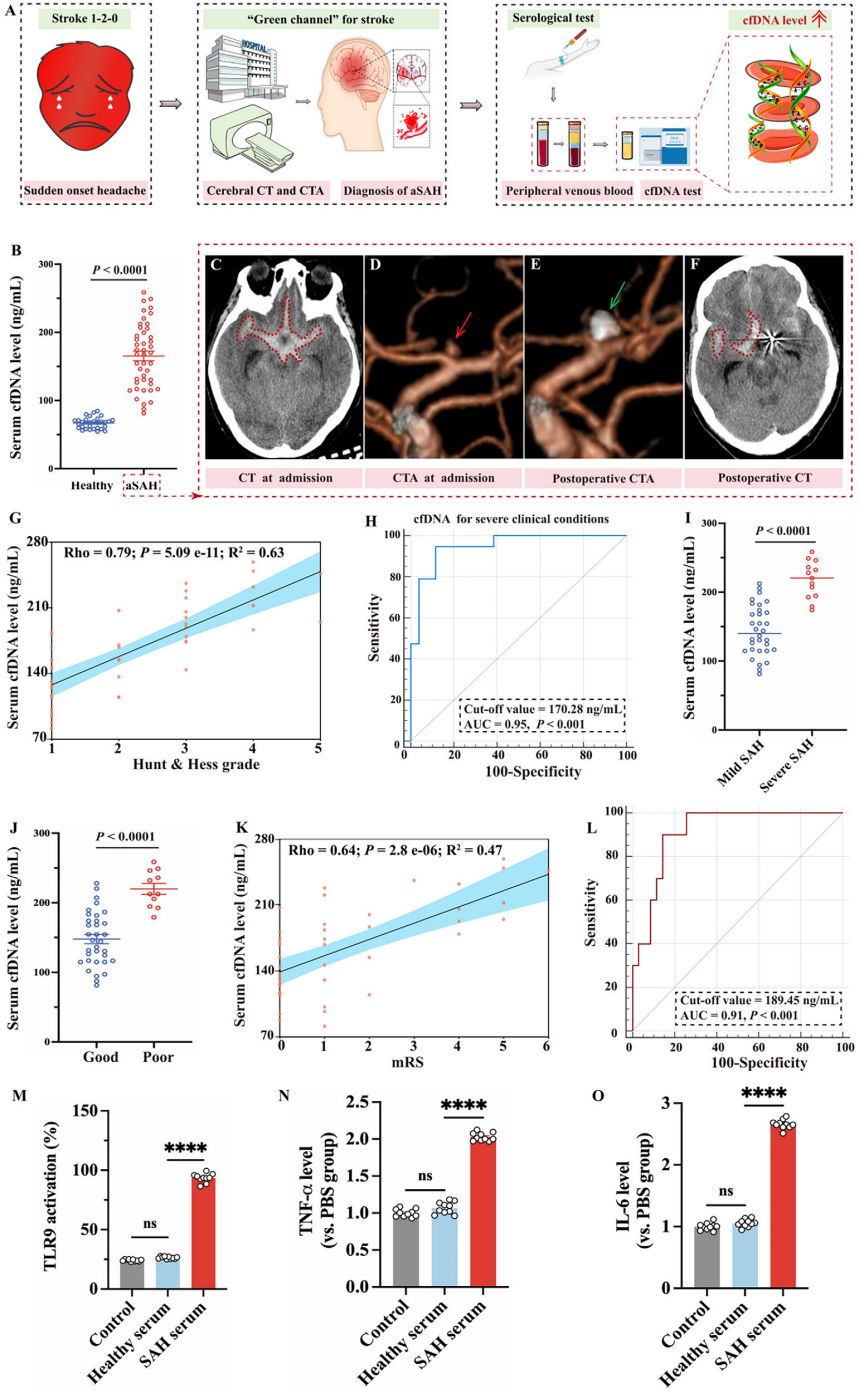
Serum cfDNA level positively correlating with the severity of brain injury and poor prognosis in patients with aSAH. A) Flow chart of cfDNA screening in clinical aSAH patients. B–F) Elevated serum cfDNA level positively correlating with the severity of aSAH and quantification comparison of serum cfDNA level between healthy controls (n = 30) and aSAH patients (n = 45). The right red dotted box represents the admission and post‐operative brain CT and CTA images of aSAH patients. The high density within the red dashed circle represents bleeding (SAH). The red arrow indicates a ruptured aneurysm, while the green arrow indicates the complete occlusion of ruptured aneurysm. G) Scatter plot of serum cfDNA levels and brain injury severity parameters, including Hunt & Hess grade and mFisher scale (n = 45). The color of the dot represents the group of subjects. The black line is the fitted regression line, and the blue shading is the 95% confidence interval (CI). (H) ROC analysis of serum cfDNA levels predicting severe clinical conditions (n = 45). I) Quantification comparison of serum cfDNA levels between mild SAH (n = 32) and severe SAH (n = 13). J) Serum cfDNA levels in aSAH patients with good (mRS 0 – 2, n = 34) and poor (mRS 3 – 6, n = 11) outcomes 3 months after discharge. K) Scatter plot of serum cfDNA levels and functional outcomes at 3 months (n = 45). The dot's color signifies the subject group, with the black line representing the regression line and the blue shading showing the 95% CI. L) ROC analysis of serum cfDNA levels predicting poor outcomes 3 months after discharge in patients with aSAH (n = 45). M) Activation of HEK‐TLR9 reporter cells by healthy human serum or aSAH patients’ serum for 24 h (n = 10). SEAP activity in the culture supernatants was quantified using the QUANTI‐Blue assay, and results were expressed as the relative absorbance percentage compared to cells treated with aSAH patients’ serum. (N, O) BV2 cells were stimulated with healthy human serum or aSAH patients’ serum for 24 h (n = 10). Supernatants were assayed for TNF‐α and IL‐6 levels via ELISA kits. Data were presented as mean ± SEM. Statistical comparisons between two groups were performed using an unpaired two‐tailed Student's t‐test, and one‐way analysis of variance (ANOVA) was applied for comparisons involving multiple groups. ns represents not significant; ^****^p < 0.0001.

In clinical practice, the diagnosis of aSAH primarily applies a combined strategy of characteristic symptoms (e.g., sudden‐onset thunderclap headaches or loss of consciousness), and radiological findings from computed tomography (CT) and computed tomography angiography (CTA) (**Figure** **2**A). To assess the severity of brain injury, aSAH patients were classified based on the Hunt & Hess grade (reflecting neurological function), and the modified Fisher (mFisher) scale (quantifying subarachnoid blood load as a marker of brain injury). Based on initial CT findings, patients were divided into mild (mFisher scale 1–2) and severe (mFisher scale 3–4) SAH, with clinical conditions similarly categorized into mild (Hunt‐Hess grade I‐II) or severe (Hunt‐Hess grade III‐V). Functional outcomes were evaluated using the modified Rankin Scale (mRS) at 3 months post‐discharge, with scores of 0–2 indicating a favorable prognosis and 3–6 indicating a poor outcome.

In patients with aSAH, serum cfDNA levels were markedly elevated compared to healthy controls (165 ± 7.08 vs 66.92 ± 1.52 ng mL^−1^, *p* < 0.001; Figure 2B–F; Table S1 and Table S2, Supporting information), underscoring its potential as a diagnostic marker for aSAH. Moreover, cfDNA levels were found to strongly correlate with brain injury severity, as evidenced by their positive correlation with Hunt & Hess grades (rho = 0.79, *p* = 5.09e‐11; Figure 2G; Figure S1A, Supporting information). The weighted univariate logistic analysis identified cfDNA as an independent predictor of severe clinical conditions (odds ratio (OR):1.08, 95% confidence interval (CI) [1.04‐1.14], *p* <0.01; Figure S2A and Table S3, Supporting information). Receiver operating characteristic (ROC) curve analysis demonstrated a high discriminatory power of cfDNA for identifying patients with severe clinical conditions, with an area under the curve (AUC) of 0.95 (*p* < 0.001; Figure 2H), reinforcing its potential as a reliable biomarker of disease severity. Similarly, cfDNA showed a robust positive correlation with the mFisher scale (rho = 0.86, *p* = 4.82e‐14). cfDNA levels were found to be significantly elevated in patients with aSAH who presented with severe SAH (217.73 ± 7.51 vs 144.11 ± 6.38 ng mL^−1^, *p* < 0.001; Figure 2I). Additionally, cfDNA emerged as an independent predictor for severe SAH (OR:1.07, 95% CI [1.03–1.13], *p* < 0.01; Figure S2B and Table S4, Supporting information). Furthermore, the cfDNA levels exhibited an AUC of 0.95 for predicting severe SAH (*p* < 0.001; Figure S1B, Supporting information). This finding serves to further validate its application in classifying the injury degrees.

Beyond its role as a biomarker, cfDNA levels also demonstrated predictive value for long‐term outcomes. At 3 months post‐discharge, aSAH patients with higher serum cfDNA levels were more likely to experience poor functional outcomes, as indicated by mRS scores (219.90 ± 7.86 versus 147.70 ± 6.63 ng mL^−1^, *p* < 0.001; Figure 2J). Furthermore, the weighted multivariable logistic regression model revealed that cfDNA independently predicted poor outcomes with an OR of 1.05 and a 95% CI of 1.01‐1.11 (*p* = 0.035; Figure S2C and Table S5, Supporting information).As demonstrated in Figure 2K, cfDNA levels were significantly correlated with a three‐month prognosis (rho = 0.64, *p* = 2.8e‐6; Figure 2K) and poor outcome (rho = 0.65, *p* = 1.16e‐6; Figure S1C, Supporting information). Additionally, cfDNA demonstrated strong predictive value for unfavorable outcomes, with an AUC of 0.91 (*p* < 0.001; Figure 2L). These findings suggest that cfDNA could reflect the severity of acute brain injury and serve as a prognostic marker for long‐term neurological recovery.

The associations between elevated cfDNA levels, severity of brain injury, and worse clinical outcomes support the hypothesis that pathological cfDNA is not merely a byproduct of cell damage but may play a role in driving the pathological process. Next, we incubate HEK‐TLR9 reporter cells with serum from healthy individuals and aSAH patients. TLR9 activation, as reflected by the activity of secreted embryonic alkaline phosphatase (SEAP), was significantly increased in response to serum from aSAH patients (Figure 2M). We then treated mouse microglial cells (BV2) with the same serum samples and observed markedly increased levels of pro‐inflammatory cytokines, including tumor necrosis factor‐α (TNF‐α) and interleukin‐6 (IL‐6) levels, in response to serum from aSAH patients (Figure 2N,O). These results indicate that the elevated serum cfDNA levels in aSAH patients contribute to TLR9 activation and subsequent inflammatory responses. Overall, the results highlight a positive correlation between serum cfDNA levels, TLR9 pathway activation, and the severity of SBI, underscoring the therapeutic potential of targeting the cfDNA‐TLR9 signaling axis in SAH.

### 2.2 Preparation and Physicochemical Characterizations of RES‐CTP@GPXP

The RES‐CTP@GPXP system was composed of two types of functional peptides, denoted RES‐CTP and GPXP. Their design principle follows (Figure 1B): 1). RES‐CTP was designed with a hydrophobic sequence (FAAVV) to drive self‐assembly, an MMP‐2 cleavable sequence (GPLGLA) to respond to the elevated MMP‐2 level in the lesion, and a cationic sequence (RKRKRKRK) to scavenge pathological cfDNA; 2). GPXP was designed with a hydrophobic sequence (FAAVV) to drive self‐assembly, a selenium‐containing glutathione peroxidase‐mimicking sequence (UKUNLN) to alleviate oxidative damage, and a neuron‐targeting sequence (LEVSRKN) to increase its uptake into damaged neurons.

**Figure 3:**
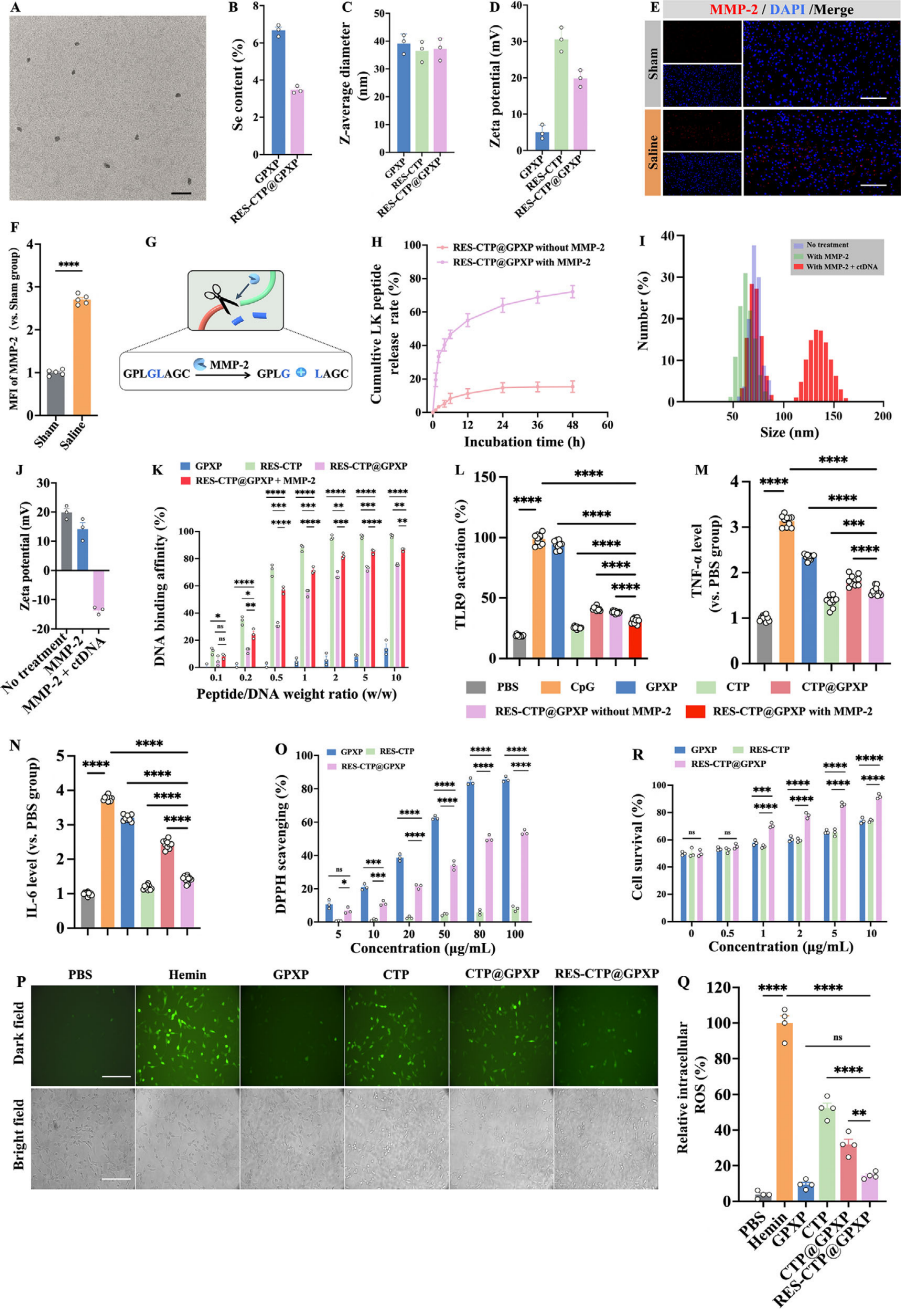
Physicochemical properties, functionality evaluations, and in vitro neuroprotective effect/safety. A) Microscopic morphology of RES‐CTP@GPXP observed by TEM. Scale bar: 200 nm. B) The Se content detected by ICP‐MS (n = 3). C,D) Z‐average diameter and zeta potential in PBS medium tested by Zeta sizer Nano ZS (n = 3). E,F) Representative immunofluorescence staining of MMP‐2 (red)/DAPI (blue) at the ipsilateral basal cortex at 24 h post‐SAH and quantitative analysis of mean fluorescence intensity (MFI) of MMP‐2. Scale bars: 100 µm (n = 5). G) GPLGLAGC peptide cleaved by MMP‐2. H) Cumulative LK release of RES‐CTP@GPXP with or without MMP‐2 treatment (n = 3). I,J) Z‐average diameter and zeta potential changes of RES‐CTP@GPXP with the sequential addition of MMP‐2 and calf thymus DNA (n = 3). K) DNA‐binding affinity of RES‐CTP@GPXP with or without MMP‐2 (n = 3). L) Activation of HEK‐TLR9 reporter cells treated with PBS, CpG, and different peptide nanoparticles for 24 h (n = 10). SEAP activity in the supernatants was quantified using the QUANTI‐Blue assay with absorbance measured at OD 620 nm. Results were presented as the percentage absorbance of cells that were treated with CpG. M,N) ELISA kits measuring TNF‐α and IL‐6 levels of BV2 cells treated with PBS, CpG, and various peptide nanoparticles 24 h (n = 10). O) DPPH scavenging efficiencies to assess total antioxidant properties (n = 3). P) DCFH‐DA probe visualizing intracellular ROS upon treatments with peptide nanoparticles. Scale bars: 100 µm. Q) Quantitative analysis of MFI reflecting intracellular ROS level (n = 4). R) Neuronal survival upon treatments with peptide nanoparticles detected using a CCK‐8 assay (n = 3). Data were presented as mean ± SEM. Statistical comparisons between two groups were performed using an unpaired two‐tailed Student's *t*‐test, and one‐way ANOVA test was applied for comparisons involving multiple groups. ns represents not significant; ^*^ *p* < 0.05, ^**^ *p* < 0.005, ^***^ *p* < 0.001, ^****^ *p* < 0.0001.

RES‐CTP and GPXP belong to amphiphilic structures, and their shared FAAVV hydrophobic sequence can drive co‐assembly in an aqueous solution. Therefore, we further examined the co‐assembly behavior of the RES‐CTP@GPXP system. It was observed that RES‐CTP and GPXP co‐assembled to form the uniform spherical particles with ≈30 nm of particle size (**Figure** **3**A). We then measured the selenium content using Inductively Coupled Plasma‐Mass Spectrometry (ICP‐MS) and found that GPXP had a relatively high selenium content of ≈6.67%, which was reduced to ≈3.47% after co‐assembling with the selenium‐free RES‐CTP (Figure 3B). These findings indicated that RES‐CTP and GPXP could effectively co‐assemble to formulate RES‐CTP@GPXP complex in an aqueous solution. Next, the surface‐charged property and particle size of RES‐CTP@GPXP were evaluated using a Zeta sizer Nano ZS after dissolving it in PBS (10 mM, pH = 7.4). As exhibited in Figure 3C,D, the results showed that GPXP had a Z‐average diameter of ≈39.13 nm and a zeta potential of ≈+5.01 mV, while RES‐CTP@GPXP had a similar Z‐average diameter and an increased zeta‐potential (≈19.83 mV) attributing to the co‐assembly with the positively‐charged RES‐CTP with high zeta potential (30.47 mV). Moreover, we found that the particle sizes and zeta potentials of RES‐CTP@GPXP exhibited good stability within 72 h, supporting their suitability for in vitro and in vivo applications (Figure S3A,B, Supporting information).

### 2.3 Functionalization Evaluations of RES‐CTP@GPXP

With the formulation of RES‐CTP@GPXP, the focus shifted to its several critical functions, mainly including MMP‐2 responsiveness, cfDNA‐binding affinity, and ROS elimination. First, we studied the MMP‐2 responsiveness of RES‐CTP@GPXP. In vitro immunofluorescence staining results showed that the MMP‐2 expression significantly increased in microglia upon hemin treatment (Figure S4A,B, Supporting information). Furthermore, SAH surgery in mice also enabled MMP‐2 levels to increase at the ipsilateral basal cortex compared to the sham group (Figure 3E,F). These results indicated the overexpression of MMP‐2 in brain lesions of SAH, providing a pathological signal for triggering the in situ split of RES‐CTP@GPXP. Our previous work proved that the peptide linkage (GPLGLAG) could be cleaved in the presence of MMP‐2 (Figure 3G).^[^ ^26^ ^]^ After incubation with MMP‐2, it was detected that the cationic sequence LAGRKRKRKRK (denoted as LK peptide) was released from the RES‐CTP@GPXP system. The cumulative LK peptide release rate reached ≈72.13% in the presence of MMP‐2, while only ≈15.40% in the absence of MMP‐2 for the RES‐CTP@GPXP system (Figure 3H). For the CTP@GPXP system, the cumulative LK peptide release rate was less than 16% regardless of the presence of MMP‐2 (Figure S5, Supporting information).

Additionally, electrospray ionization mass spectrometry (ESI‐MS) was performed to detect the released LK peptide after dialysis and lyophilization at 48 h of MMP‐2 incubation (Figure S6, Supporting information). This indicated that the MMP‐2 could induce the cleavage of the GPLGLAG linkage and the release of cationic LK peptide. Furthermore, we also monitored changes in colloid properties after adding MMP‐2 and found that Z‐average diameter was reduced from ≈37.15 to ≈25.05 nm, and zeta potential was also decreased from ≈+19.83 to ≈+14.2 mV (Figure 3J). This suggests that the LK peptide detached from the co‐assembled system RES‐CTP@GPXP, decreasing its particle size and surface positive‐charge. These results support that the RES‐CTP@GPXP can remain stable under normal conditions and then split into the LK peptide and remaining GPXP under pathological MMP‐2 conditions.

Subsequently, the DNA binding affinity of the RES‐CTP@GPXP system was investigated through the competitive binding of double‐stranded DNA (dsDNA) with the PicoGreen agent. It was observed that the DNA binding rate of RES‐CTP@GPXP increased with the peptide/DNA weight ratio and could achieve ≈54.94% at weight ratio of 1.0, and ≈75.58% at weight ratio of 10.0 (Figure 3K). However, its DNA binding capacity couldn't avoid the weaker fate in comparison with the RES‐CTP group. This was mainly because the neutral GPXP could dilute the positive‐charges from RES‐CTP after co‐assembly. Fortunately, the cationic LK peptide could be released from the co‐assembled system to recover its positively‐charged effect upon incubation with MMP‐2, thereby enabling DNA binding capacity to be further enhanced. In addition, we further investigated the DNA scavenging property. As shown in Figure 3J, after MMP‐2 incubation, the calf thymus DNA (ctDNA, a typical dsDNA) was sequentially added with the Z‐average diameter increasing to ≈91.68 nm and zeta potential reducing to −‐13.70 mV. This illustrates that dsDNA could be scavenged by the cationic LK peptide via electrostatic absorptions to form nanosized complexes, which is beneficial in inhibiting the binding between cfDNA and TLR9.

As established in the literatures, cfDNA—mainly including CpG oligonucleotide—acts as a potent DAMP that can bind with TLR9 to activate TLR9 and downstream NF‐κB signaling to initiate inflammatory responses.^[^ ^12^, ^14^, ^15^, ^17^, ^18^, ^19^, ^20^ ^]^ To confirm this mechanism, we used CpG to stimulate HEK‐TLR9 reporter cells and BV2 microglia cells respectively, finding the significant TLR9 activation (Figure 3L) and the increased secretions of pro‐inflammatory cytokines TNF‐α and IL‐6 (Figure 3M,N). Then, further addition of the peptide nanoparticle CTP only with cfDNA‐scavenging property and without ROS‐mitigation property could inhibit TLR9 activation. This mechanism indicates that peptide nanoparticle CTP can electrostatically bind with CpG (Figure 3K), and inhibit the binding between CpG and TLR9, thereby inhibiting the TLR9 activation and downstream NF‐κB signaling, which is consistent with other similar papers.^[^ ^12^, ^13^, ^14^, ^16^, ^24^ ^]^

Next, we aimed to assess the antioxidant potential of RES‐CTP@GPXP. In this section, the diphenyl‐1‐picrylhydrazyl (DPPH) assay was performed to evaluate the total antioxidant capacity. As exhibited in Figure 3O, the RES‐CTP@GPXP could inhibit the production of DPPH radicals to varying degrees. RES‐CTP@GPXP exhibited > 50% scavenging efficiency at ≈80 µg mL^−1^ for DPPH, which overall inherited the antioxidant property from the GPXP as a positive control. Besides, the 2, 2‐azino‐bis (3‐ethylbenzothiazoline‐6‐sulphonic acid) (ABTS) assay was also carried out to support this claim (Figure S7, Supporting information). Furthermore, we measured the antioxidant performance of RES‐CTP@GPXP in cells using dichlorodihydrofluorescein diacetate (DCFH‐DA)assay. According to our previous work,^[^ ^27^ ^]^ a cell model of hemorrhagic process‐induced neuron injury was established by treating mouse hippocampal neuronal cells (HT22 cells) with 60 µM of hemin, which resulted in the generation of a large amount of ROS. The RES‐CTP@GPXP treatment effectively downregulated the intracellular ROS level from ≈100% to ≈14.43%, which could largely alleviate the oxidative stress in HT22 cells (Figure 3P,Q).

Afterward, we investigated whether ROS clearance could inhibit TLR9/NF‐κB signaling. Excessive ROS production plays a central role in driving neuroinflammation following SAH, particularly through activation of the ROS–NF‐κB signaling axis.^[^ ^27^, ^28^, ^29^ ^]^ First, our result confirmed that the peptide nanoparticle GPXP (a selenium‐containing glutathione peroxidase‐mimicking) only with ROS mitigation property and without cfDNA‐scavenging property couldn't obviously inhibit TLR9 activation (Figure 3L), but could inhibit the secretions of TNF‐α and IL‐6 (Figure 3M,N). This indicated that peptide nanoparticle GPXP could inhibit NF‐κB signaling probably via ROS–NF‐κB axis rather than TLR9 pathway. Next, GPX4 is known as a potent antioxidant molecule for redox‐regulatory function. Immunohistochemistry staining revealed that GPXP treatment upregulated the expression of GPX4 (Figure S8, Supporting information). RES‐CTP@GPXP and CTP@GPXP treatment also increased the levels of GPX4, indicating that they maintain the antioxidant function of GPXP. Furthermore, in vitro experiments also revealed that GPXP treatment enhanced the cellular antioxidant defense system, significantly reducing intracellular ROS levels (Figure 3P,Q). These results indicated the mechanism that the peptide nanoparticle GPXP upregulated GPX4 to mitigate ROS level, thereby alleviating NF‐κB signaling and inflammation.^[^ ^9^, ^27^, ^28^, ^29^ ^]^

Together, our final peptide drug RES‐CTP@GPXP containing CTP and GPXP acts as dual‐mechanisms: 1) capturing pathological cfDNA to block cfDNA‐TLR9 binding, thereby inhibiting TLR9 activation; 2) reducing local ROS level via GPX4 upregulation. The two effects synergistically inhibited NF‐κB signaling, alleviated neuroinflammation, and acquired good neuroprotective effect (Figure 3K‐R). The key functional attributes of RES‐CTP@GPXP, including MMP‐2 responsiveness, high cfDNA‐binding affinity, and potent ROS‐scavenging ability, support its in situ proliferative behavior and dual‐scavenge pathological cfDNA/ROS in further in vivo experiments.

### 2.4 In Vitro evaluations of Neuroprotective Effect and Safety

To evaluate the neuroprotective effect and safety of RES‐CTP@GPXP, in vitro cell experiments were preliminarily carried out. First, based on our previous work,^[^ ^27^ ^]^ a cell model of SAH was created by treating HT22 with hemin treatment at a concentration of 60 µM, where cell viability was reduced to ≈50.07%. Then, to illustrate the superiority of the MMP‐2 mediated in situ proliferation strategy, we applied single‐target treatment groups (individual RES‐CTP, and GPXP), the simple‐combination of CTP@GPXP without MMP‐2 responsiveness as a conventional multi‐target treatment group, and RES‐CTP@GPXP as a flexible multi‐target treatment group to treat HT22. Results showed that RES‐CTP@GPXP treatment saved cell survival with the highest survival rate of ≈91.81% at 10 µg/mL (Figure 3R), whose in vitro neuroprotective effect was stronger than other treatments, attributing to their more effective dual‐scavenging of cfDNA/ROS. Furthermore, a standard Cell counting Kit‐8 (CCK‐8) assay was also used to evaluate their cytotoxicity. Various concentrations of CTP@GPXP and RES‐CTP@GPXP were cultured with neuro‐glial unit‐related cells, including HT22 cells and BV2 cells (Figure S9A,B, Supporting information). After 24 h of incubation, these cells demonstrated more than 95% viability even when they were exposed to a high concentration of 50 µg mL^−1^. Therefore, RES‐CTP@GPXP displayed a potent effect in inhibiting neuronal cell death. Nonetheless, PAMAM‐G3, a traditional material for scavenging cfDNA, exhibited increased cytotoxicity. These results highlighted the safety and therapeutic potential of the RES‐CTP@GPXP system.

### 2.5 Intranasal Delivery of RES‐CTP@GPXP to the Brain Following SAH

The blood‐brain barrier (BBB) remains a critical challenge in the development of therapeutic targeting center nervous system (CNS) diseases, primarily owing to the presence of tight junctions between brain microvascular endothelial cells that restrict molecular penetration.^[^ ^30^ ^]^ Although SAH can transiently disrupt the integrity of the BBB and allow limited drug penetration into the brain, this window of increased permeability is short‐lived. Consequently, large molecules and nanomedicine‐based delivery systems administered through conventional routes, such as intravenous injection, still face significant challenges in crossing the BBB. This often necessitates the use of additional strategies such as surface functionalization to enhance permeability.^[^ ^31^, ^32^ ^]^

In contrast, the nose‐to‐brain pathway has gained increasing attention as a promising, non‐invasive, and efficient route for CNS drug delivery. This approach primarily utilizes the olfactory and trigeminal nerve pathways to enable direct drug transport to the brain, effectively bypassing the BBB.^[^ ^33^, ^34^, ^35^ ^]^ The underlying anatomical basis lies in the location of the olfactory epithelium at the roof of the nasal cavity, which is in close proximity to the olfactory nerve and directly connected to the olfactory bulb. This unique neuroanatomical access renders intranasal administration an attractive and efficient strategy for brain‐targeted therapies, with growing potential for clinical translation.^[^ ^33^, ^35^ ^]^

**Figure 4:**
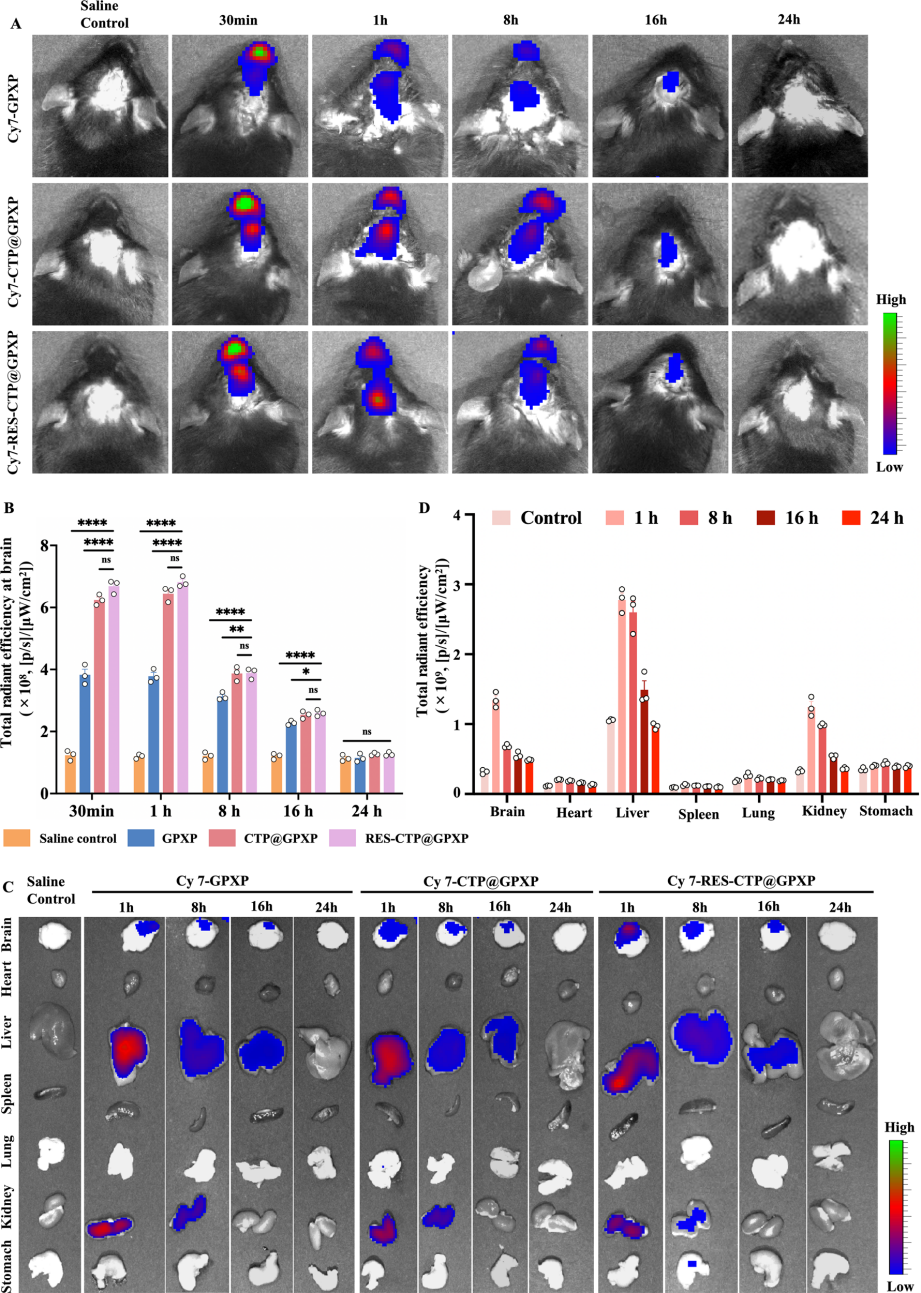
Intranasal delivery of peptide nanoparticles into brain following SAH. A) In vivo fluorescence imaging was performed to assess brain accumulation in SAH mice following intranasal administration of Cy7‐labeled GPXP, CTP@GPXP, and RES‐CTP@GPXP nanoparticles at different time points. B) Quantitative analysis of total radiant efficiency at the brain (n = 3). (C) *Ex vivo* fluorescence imaging of isolated brain tissues and other major organs following administration of the different peptide nanoparticles at different time points. D) Quantitative *ex vivo* analysis of total radiant efficiency in isolated brain tissues and other major organs after RES‐CTP@GPXP treatment (n = 3). Data were presented as mean ± SEM. Statistical comparisons between two groups were conducted using an unpaired two‐tailed Student's t‐test, and one‐way ANOVA test was applied for comparisons among multiple groups. ns represents not significant; *p* > 0.05, ^*^ *p* < 0.05, ^**^ *p* < 0.005, ^****^ *p* < 0.0001.

In our study, the Cy7‐labeled peptide nanoparticles, including Cy7‐GPXP, Cy7‐CTP@GPXP, and Cy7‐RES‐CTP@GPXP, were intranasally administered to SAH mice and tracked using an in vivo fluorescence imaging system (IVIS). In the Cy7 ‐RES‐CTP@GPXP group, fluorescence signals were observed in the brain within 30 min of administration, which significantly intensified by 1 h and persisted for at least 16 h. The fluorescence intensity of RES‐CTP@GPXP reached its peak during this period, indicating effective and sustained brain accumulation (**Figure** **4**A,B). Notably, the nose to the brain transfer efficiency of Cy7 ‐RES‐CTP@GPXP and Cy7‐CTP@GPXP was comparable and significantly higher than that of Cy7‐GPXP, suggesting that co‐assembly with CTP enhances brain‐targeting efficiency.

The enhanced intranasal delivery of Cy7‐RES‐CTP@GPXP highlights its potential to rapidly achieve therapeutic concentrations in the brain, making it a promising candidate for emergency treatment of SAH. *Ex vivo* fluorescence imaging of major organs, including the liver and kidneys, revealed that Cy7‐RES‐CTP@GPXP, Cy7‐CTP@GPXP, and Cy7‐GPXP were also distributed in these organs, likely due to partial absorption into the systemic circulation through the nasal mucosal capillary network (Figure 4C,D; Figure S10A,B, Supporting information). However, the fluorescence in peripheral organs decreased significantly by 24 h, indicating clearance through hepatic metabolism or renal excretion.

Collectively, these findings demonstrate that intranasal administration of Cy7‐RES‐CTP@GPXP enables rapid, efficient, and sustained brain delivery while minimizing prolonged systemic exposure. This delivery strategy offers strong therapeutic potential for acute neurological disorders such as SAH, where timely and targeted drug intervention is essential.

### 2.6 In Vivo Evaluation of Regulating the Dysfunctions of Neuro‐Glial Units by Inhibiting Neuroinflammation and Oxidative Stress

Dysfunctions of the neuro‐glial unit are a major pathological hallmark of SBI, characterized by severe neuroinflammation and oxidative stress. These two processes are intricately interdependent, forming a vicious cycle that exacerbates neuronal damage and complicates therapeutic intervention.^[^ ^10^ ^]^ During the acute phase of SAH, resident immune cells (e.g., microglia and astrocytes, the principal immune effector cells in the brain, accumulated in close proximity to injury sites), were excessively activated to release cytotoxic pro‐inflammatory factors including IL‐6, TNF‐α, and ROS. This cascading event induced neuroinflammation and oxidative damage, leading to neuronal injury. Conversely, the inflammatory cytokines and oxidative mediators created a feedback loop that enhanced glial cell activation and further disrupted the neuro‐glial interactions, leading to persistent neuroinflammation and oxidative stress, ultimately exacerbating neurological outcomes.^[^ ^8^, ^9^ ^]^

**Figure 5:**
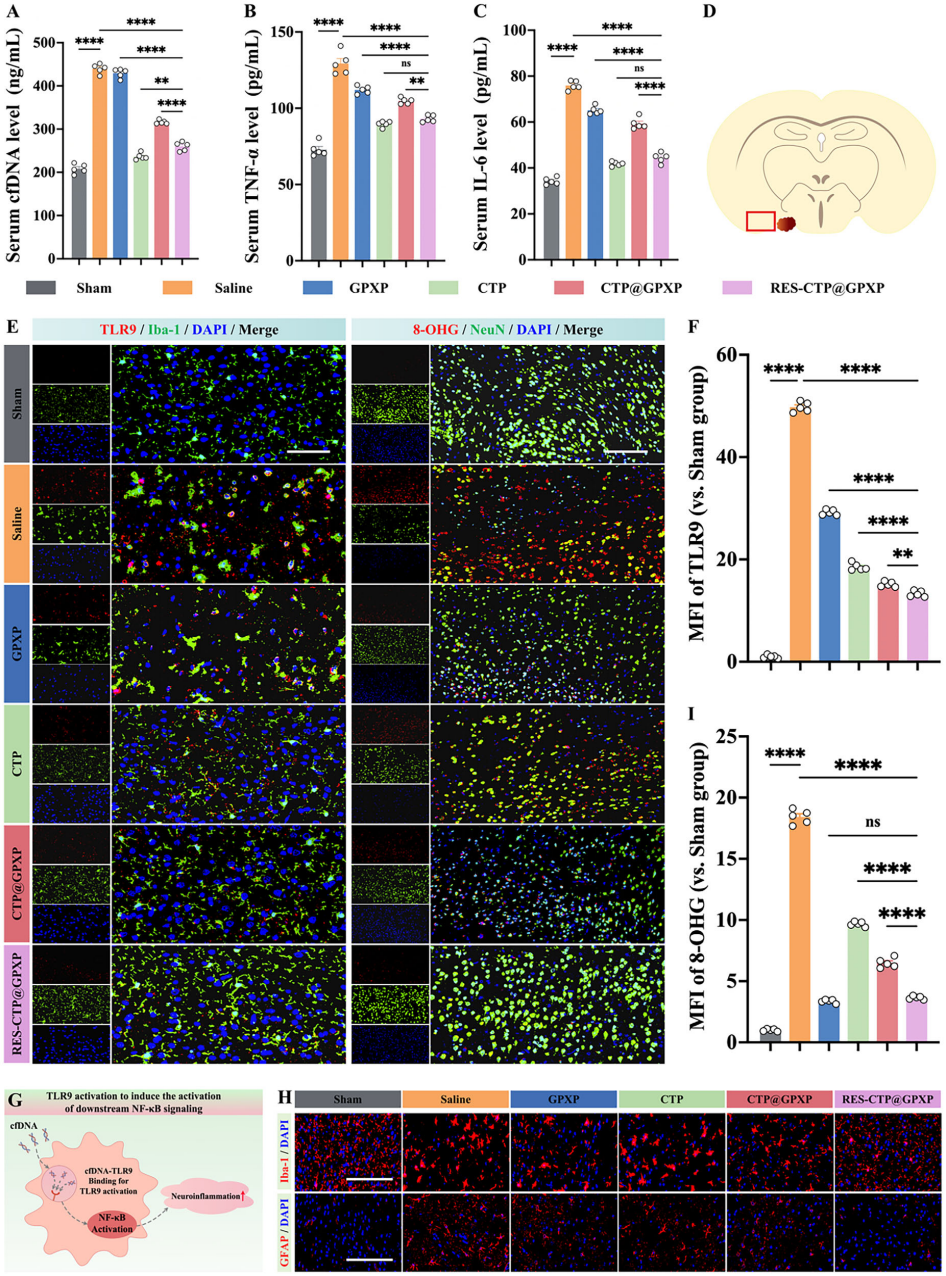
In vivo inhibitory effects on neuroinflammation and oxidative stress following SAH. A) CfDNA levels in serum upon various treatments at 24 h post of SAH. (n = 5). B) TNF‐α levels in serum upon various treatments at 24 h post of SAH. (n = 5). C) IL‐6 levels in serum upon various treatments at 24 h post of SAH. (n = 5). D) A red mark in the coronal section of brain indicating area used for microphotographs. E) Representative immunofluorescence images showing the expression of TLR9 (red)/Iba‐1 (green)/DAPI (blue) and 8‐OHG (red)/NeuN (green)/DAPI (blue) at the ipsilateral basal cortex at 24 h following SAH. Scale bars: 100 µm. F) Quantification of MFI of TLR9 expression. (n = 5). G) Schematic illustration of TLR9 activation to induce the activation of downstream NF‐κB signaling pathway. H) Representative immunofluorescence staining of Iba‐1 (red)/DAPI (blue) and GFAP (red)/DAPI (blue) at the ipsilateral basal cortex at day 3 following SAH, indicating activation of microglia and astrocytes. Scale bars: 50 µm. (n = 5). I) Quantitative analysis of MFI for 8‐OHG expression (n = 5). Data were presented as mean ± SEM. Statistical comparisons between two groups were conducted using an unpaired two‐tailed Student's *t*‐test, and one‐way ANOVA test was applied for comparisons involving multiple groups. ns represents not significant; ^*^ *p* < 0.05, ^**^ *p*< 0.005, ^***^ *p* < 0.001, ^****^ *p* < 0.0001.

Recent studies have emphasized the crucial role of TLR9 in sensing DAMP, such as cfDNA, which were also elevated in other inflammatory diseases.^[^ ^12^, ^13^, ^14^, ^15^, ^17^, ^21^, ^36^ ^]^ CfDNA can directly activate TLR9 and trigger downstream the NF‐κB signaling, thereby amplifying inflammatory cascades.^[^ ^14^, ^15^, ^17^, ^37^ ^]^ The occurrence of SAH contributed to an increase in serum cfDNA levels in clinical patients and experimental mice, where the cfDNA levels correlated positively with the magnitude of brain injury (Figures 2B–I and **5**A). Correspondingly, serum concentrations of the pro‐inflammatory cytokines TNF‐α and IL‐6 were also significantly increased (Figure 5B,C), suggesting the involvement of cfDNA‐mediated inflammatory signaling. Hence, it was speculated that the newly identified pattern of cfDNA‐driven neuroinflammation might be triggered.

To investigate this hypothesis, we examined brain tissue from the ipsilateral basal cortex in mice with SAH. Upon immunofluorescence staining, TLR9 was found to be predominantly activated in microglia within the lesion site in the SAH model compared to the sham group, further supporting cfDNA‐driven microglial activation and neuroinflammation (Figure 5D–G). Additionally, we observed significant glial activation, as evidenced by the increased numbers of Iba‐1‐ and GFAP‐positive cells (Figure 5H). Morphologically, activated microglia displayed enlarged cell bodies with shortened processes, while reactive astrocytes exhibited hypertrophy and extended processes. Together, these findings confirm that pathological cfDNA contributes to post‐SAH neuroinflammation via TLR9/NF‐κB signaling.

Among various treatment strategies, the RES‐CTP@GPXP system showed superior efficacy in mitigating neuroinflammation. By lowing pathological cfDNA levels in serum and inhibiting the TLR9/NF‐κB signaling axis, it effectively suppressed glial cell activations and downregulated TNF‐α and IL‐6 expression (Figure 5B,C,G), outperforming the CTP@GPXP treatment.

Next, we further explored the inhibitory effects on oxidative stress. Oxidative stress is a critical factor in neuronal injury, contributing to both the initiation and exacerbation of neuroinflammation.^[^ ^7^, ^16^, ^27^, ^38^ ^]^ Following SAH, neuronal oxidative damage was evidenced by elevated 8‐hydroxyguanosine (8‐OHG, a DNA/RNA oxidation biomarker) levels, predominantly localized in the affected neurons (Figure 5E,I). The RES‐CTP@GPXP treatment effectively alleviated this oxidative damage, owing to the ROS‐scavenging function of GPXP peptide, which was better than CTP@GPXP treatment.

Mechanistically, RES‐CTP@GPXP system undergoes MMP‐2 mediated in situ proliferation at the lesion sites, dissociating into its individual components—CTP and GPXP components. Among them, CTP functions to scavenge pathological cfDNA and suppress TLR9‐mediated neuroinflammation, while the GPXP targets neurons to mitigate oxidative damage. This flexible and synergistic strategy effectively addresses both major pathological components of SBI, facilitating the restoration of neuro‐glial homeostasis and offering a promising therapeutic strategy for SAH.

### 2.7 In Vivo Evaluation of Regulating the Dysfunctions of Neuro‐Glial Unit by Inhibiting Programmed Cell Death

**Figure 6:**
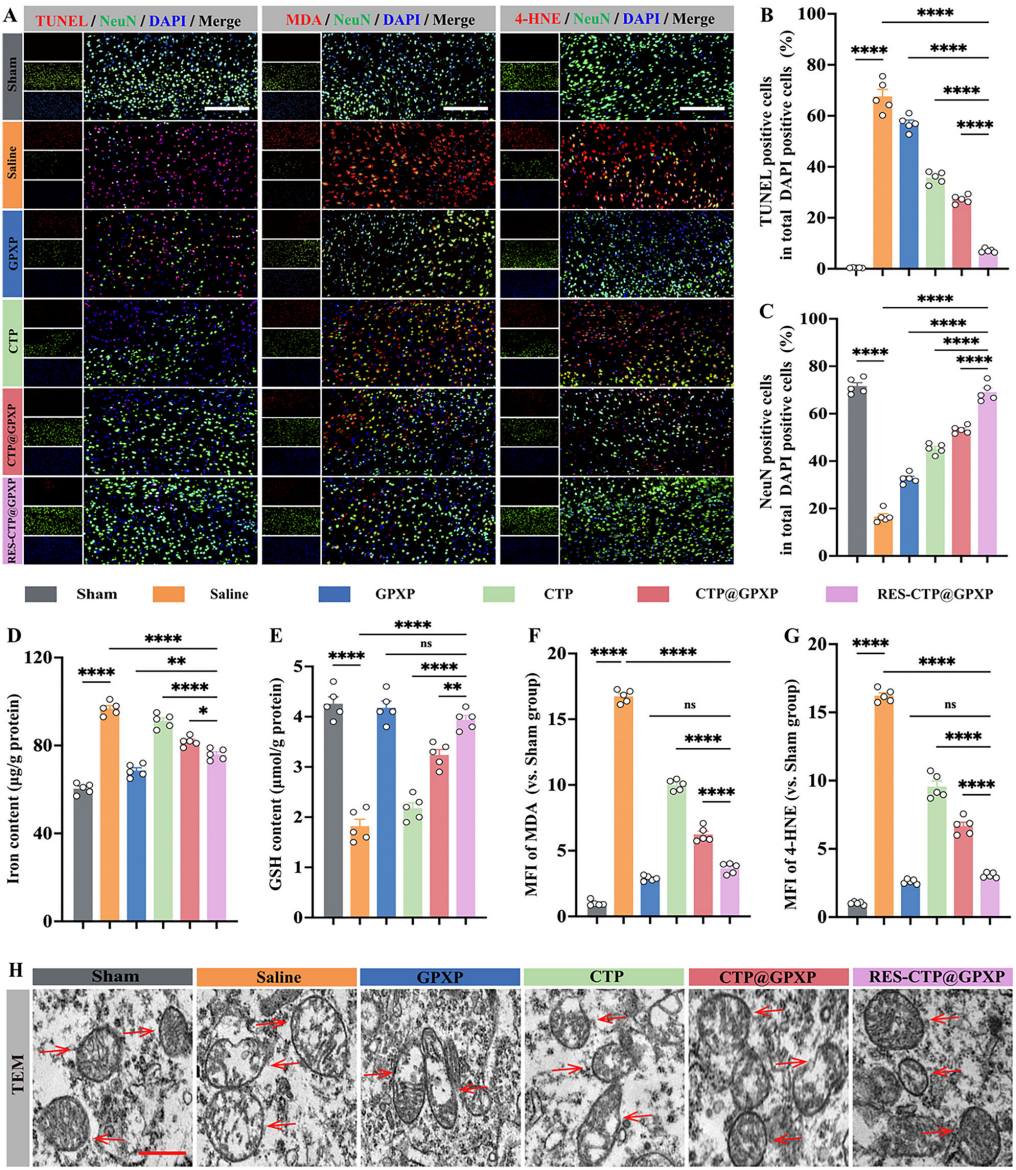
In vivo inhibitory effects on neuronal apoptosis and ferroptosis following SAH. A) Representative immunofluorescence staining of TUNEL (red)/NeuN (green)/DAPI (blue), MDA (red)/NeuN(green)/DAPI (blue), and 4‐HNE (red)/NeuN(green)/DAPI (blue) at the ipsilateral basal cortex on day 3 after SAH. Scale bars: 100 µm. B) Quantitative analysis of TUNEL‐positive cells in the total DAPI‐positive cells. (n = 5). C) Quantitative analysis of NeuN‐positive cells in the total DAPI‐positive cells. (n = 5). D) Measurement of iron content at the ipsilateral basal cortex using an iron assay kit at day 3 post‐SAH (n = 5). E) Quantification of GSH levels using a GSH assay kit at the ipsilateral basal cortex on day 3 post‐SAH (n = 5). F,G) Quantitative analysis of MFI of MDA and 4‐HNE. (n = 5). H) Mitochondrial morphology observed by TEM at the ipsilateral basal cortex on day 3 following SAH. Scale bars: 2 µm. Data were presented as mean ± SEM. Statistical comparisons between two groups were conducted using an unpaired two‐tailed Student's t‐test, and one‐way ANOVA test was employed for comparisons among multiple groups. ns: not significant; ^*^ *p* < 0.05, ^**^ *p* < 0.005, ^***^ *p* < 0.001, ^****^ *p* < 0.0001.

Afterward, we further explored the potential inhibitory effects on programmed neuronal cell death. SAH induced both neuroinflammation and oxidative damage, which could drive different types of programmed cell death, with neuronal apoptosis and ferroptosis emerging as particularly important roles.^[^ ^38^, ^39^ ^]^ First, the NeuN/TUNEL staining was conducted on day 3 post‐SAH, revealing a marked increase in TUNEL‐positive neurons in the saline‐treated group, indicative of extensive neuronal apoptosis within the ipsilateral basal cortex (**Figure** **6**A–C). However, RES‐CTP@GPXP treatment substantially reduced the proportion of apoptotic cells in comparison with other treatment groups. This finding supports that RES‐CTP@GPXP can effectively attenuate neuronal apoptosis following SAH.

Ferroptosis is mainly characterized by iron‐dependent lipid peroxidation and mitochondrial abnormalities, including outer membrane rupture and reduced cristae.^[^ ^27^ ^]^ This type of programmed cell death was increasingly recognized as a major contributor to neuronal injury in the context of SAH, driven by dysregulated iron metabolism and excessive oxidative stress.^[^ ^38^, ^40^ ^]^ Hence, we investigated the typical characteristics of ferroptosis, which included increased iron level and reduced glutathione (GSH)level, implying that cellular antioxidant defense was impaired.^[^ ^41^, ^42^, ^43^ ^]^ Compared with sham group, SAH‐induced ferroptosis resulted in increased iron and decreased GSH levels following SAH (Figure 6D,E). Besides, malondialdehyde (MDA) and 4‐Hydroxynonenal (4‐HNE), important markers of ferroptosis, were by‐products of lipid peroxidation, and their accumulation indicated the increased oxidative damage and lipid peroxidation, signifying ferroptosis. As shown in Figure 6A,F,G, the saline group largely increased the MDA and 4‐HNE levels. Furthermore, transmission electron microscopy (TEM) imaging revealed notable ultrastructural alterations in neuronal mitochondria following SAH, characterized by disrupted mitochondrial membranes and a significant loss of cristae. These morphological changes were consistent with the induction of neuronal ferroptosis (Figure 6H). Following treatment, RES‐CTP@GPXP exhibited a robust ability to restore these abnormal changes, whose inhibitory effect on neuronal ferroptosis was better than the CTP@GPXP group without the MMP‐2 mediated in situ proliferation action.

### 2.8 In Vivo Evaluation of Improving Brain Injury and Neurofunctions

Based upon our previous findings, the flexible multi‐target peptide nanoparticle, RES‐CTP@GPXP, exhibited strong therapeutic potential in alleviating neuroinflammation and oxidative damage, as well as inhibiting neuronal apoptosis and ferroptosis following SAH. These protective effects are largely attributed to its highly efficient dual‐scavenging capability against cfDNA and ROS – two key pathological mediators that contribute significantly to SBI.

**Figure 7:**
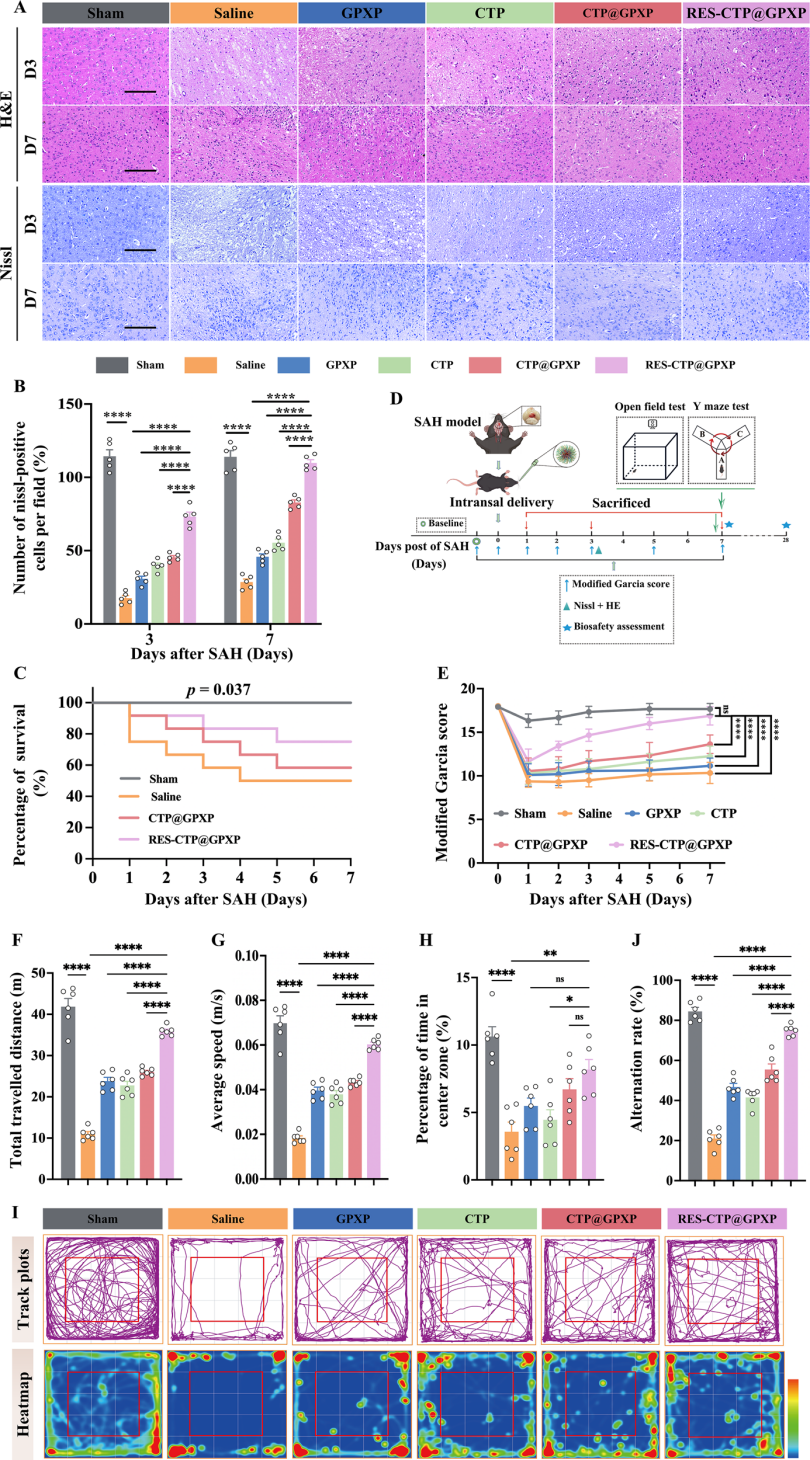
In vivo evaluation of improving brain injury and neurofunctions following SAH. A) Representative H&E and Nissl staining at the ipsilateral basal cortex on days 3 and 7 following SAH observing changes in neuroanatomical structures. Scale bars: 100 µm. B) Quantification of Nissl‐positive cells at the ipsilateral cortex by Nissl staining on days 3 and 7 post‐SAH (n = 5). C) Kaplan–Meier survival curve of mice upon various treatments (n = 12). D) Schematic illustration of the timeline of animal experiments, including the mGS and behavioral tests. E) Quantitative sensorimotor function assessment using mGS (n = 12). F–H) Open field test parameters measured on day 7 following SAH (n = 6), including total distance traveled (F), average speed (G), percentage of time in center zone (H). I) Representative track plots and corresponding heatmaps during the open field test. J) Spontaneous alteration rate in Y maze test on day 7 following SAH (n = 6). Data were presented as mean ± SEM. Statistical comparisons between two groups were performed using an unpaired two‐tailed Student's t‐test, and one‐way ANOVA test was applied for comparisons among multiple groups. ns: not significant; *p* > 0.05, ^*^ *p* < 0.05, ^**^ *p* < 0.005, ^***^ *p* < 0.001, ^****^ *p* < 0.0001.

To further evaluate its neuroprotective efficacy, we performed histopathological analyses using hematoxylin & eosin (H&E) and Nissl staining (**Figure** **7**A). In saline‐treated SAH mice, extensive neuroanatomical abnormalities were observed, including disrupted neuronal layering, cytoplasmic vacuolation, and nuclear pyknosis. In contrast, mice treated with RES‐CTP@GPXP showed notable restoration of normal neuroanatomical structure and reduced immune cell infiltration by day 7 (Figure 7A), indicating effective attenuation of neuroinflammation. Nissl staining further confirmed the preservation of neuronal integrity. While the number of Nissl‐positive neurons in the basal cortex of saline‐treated mice dropped to fewer than 30 by day 7, RES‐CTP@GPXP treatment increased this number to ≈73 on day 3 and 109 on day 7 (Figure 7A,B). These results suggest that RES‐CTP@GPXP significantly mitigates neuronal loss and promotes structural recovery following SAH. Moreover, Kaplan‐Meier survival analysis demonstrated that RES‐CTP@GPXP treatment significantly improved 7‐day survival rates compared to controls (Figure 7C), underscoring its potential to enhance clinical outcomes in SAH through multifaceted neuroprotection.

Next, we further conducted neurological assessments and behavioral tests to evaluate the recovery of neurological function following various treatments after SAH (Figure 7D). Sensorimotor function was first assessed using the modified Garcia score (mGS). Mice in the saline‐treated group exhibited significantly lower mGS, indicating pronounced sensorimotor deficits induced by SAH (Figure 7E). In contrast, treatment with RES‐CTP@GPXP significantly improved neurological function, with mGS increasing to ≈16, suggesting a marked recovery of sensorimotor abilities and overall neurological performance.

Behavioral assessments were also conducted, including the open field test and Y‐maze spontaneous alternation task. Open field test was used to assess motor function and physical activity by measuring the total distance traveled and mean velocity. Mice treated with RES‐CTP@GPXP exhibited a significant increase in total distance traveled and mean velocity, indicating enhanced locomotor activity and recovery of physical function compared to other treatment groups (Figure 7F,G). In addition, anxiety‐like behaviors were assessed by the percentage of time spent in the center zone of the open field. Mice receiving RES‐CTP@GPXP treatment spent more time spent in the center zone (Figure 7H,I), suggesting reduced anxiety‐like behavior. Furthermore, spatial working memory, a commonly impaired neurofunction post‐SAH, was assessed using the Y‐maze spontaneous alternation task. Mice treated with RES‐CTP@GPXP demonstrated a significantly higher alternation rate, reflecting improved working memory function (Figure 7J; Figure S11, Supporting information). Collectively, these findings highlight the robust neurobehavioral benefits of RES‐CTP@GPXP as an advanced multi‐target therapeutic platform for improving functional recovery after SAH.

### 2.9 Transcriptomic Profiling Reveals that RES‐CTP@GPXP Modulates Neuro‐Glial Unit Homeostasis Through Multi‐Pathway Regulation

**Figure 8:**
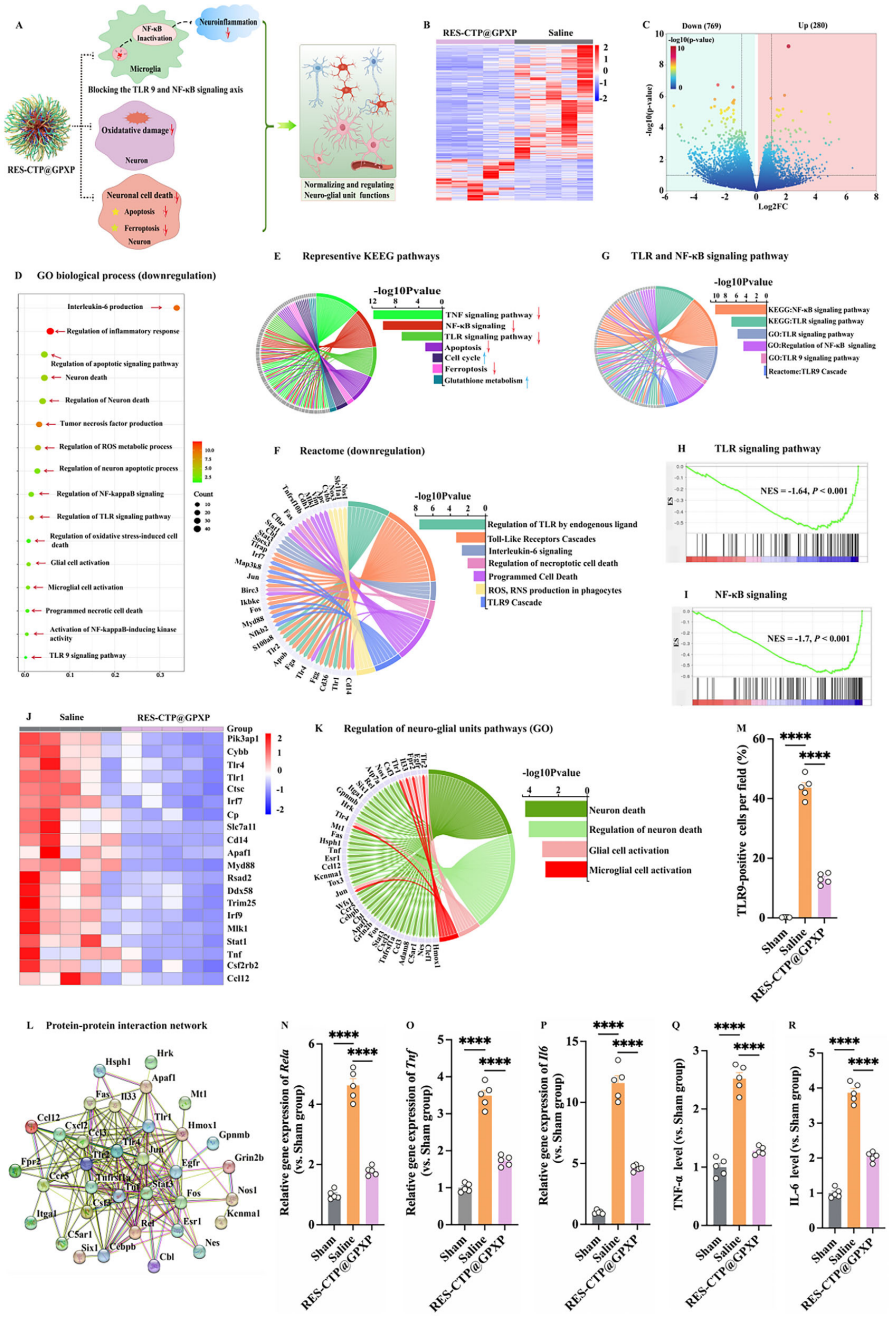
Bulk RNA sequencing analysis following RES‐CTP@GPXP treatment. A) Illustration of the therapeutic mechanism of RES‐CTP@GPXP. B) Hierarchical clustering of DEGs between RES‐CTP@GPXP treatment and saline treatment groups. The rows represented the degree of gene clustering, and the columns represented individual repeats (n = 5). Red represented the upregulated genes, and blue represented the downregulated genes. C) Volcano plots for all DEGs between RES‐CTP@GPXP treatment and saline treatment groups (n = 5). Dots represented DEGs (|log_2_FC|≥1 and *P* < 0.05). D) Enrichment analysis of GO term between RES‐CTP@GPXP treatment and saline treatment. E) Representative KEEG enrichment pathway. Chord diagram: The right semicircle represented KEGG terms, and the left semicircle represented DEGs. The color map represented the fold‐change of genes, and color bands connected genes to a specific KEGG term. The horizontal axis represented the −log10(p‐value) of the pathway and the vertical axis represents the KEGG terms. Red arrows represented the downregulated signaling pathways, and blue arrows represented the upregulated signaling pathways. F) Reactome enrichment pathways (downregulation). Chord diagram: The right semicircle represented Reactome terms, and the left semicircle represented DEGs. The color map represented the fold‐change of genes, and color bands connected genes to a specific Reactome term. The horizontal axis represented the −log10(p‐value) of the pathway and the vertical axis represents the Reactome terms. G) Enrichment analysis of TLR and NF‐κB signaling pathway from GO, KEGG, and Reactome databases. H–I) GSEA enrichment plots reveal inhibitory states of Toll‐like receptor signaling pathway, NF‐κB signaling. J) Heatmap of the representative genes from relevant pathways. Red represented the upregulated genes, and blue represented the downregulated genes. K) GO enrichment pathway for regulating neuro‐glial unit pathways. Chord diagram: The right semicircle represented GO terms, and the left semicircle represented DEGs. The color map represented the fold‐change of genes, and color bands connected genes to a specific GO term. The horizontal axis represented the −log10(p‐value) of the pathway and the vertical axis represents the GO terms. L) Protein‐protein interaction network for regulating the neuro‐glial unit relevant pathways. M) Quantitative analysis of the TLR9 positive cell number at the ipsilateral basal cortex on day 3 post‐SAH(n = 5). N–P) Relative gene expressions of *Rela, Tnf, and Il6* at the ipsilateral basal cortex on day 3 post‐SAH (n = 5). Q,R) Levels of proinflammatory cytokine TNF‐α and IL‐6 upon RES‐CTP@GPXP treatment at the ipsilateral basal cortex on day 3 following SAH (n = 5). Data were presented as mean ± SEM and one‐way ANOVA test was applied for comparisons involving multiple groups. ^****^ *p* < 0.0001. ES, enrichment score; NES, normalized enrichment score.

The neuro‐glial unit serves as a fundamental structural and functional hub in the CNS, orchestrating interactions between neurons and glial cells to maintain brain homeostasis. Its disruption is a pathological hallmark of SBI, where a vicious cycle of neuroinflammation and oxidative stress leads to progressive neuronal damage. Integrated in vitro and in vivo results confirmed that RES‐CTP@GPXP exerts dual therapeutic actions—scavenging cfDNA and mitigating ROS—which collectively inhibit TLR9/NF‐κB signaling and reduce the release of proinflammatory cytokines (TNF‐α, IL‐6), as well as alleviate neuronal oxidative stress. Mechanistically, RES‐CTP@GPXP targets three interlinked pathological hallmarks of SBI: neuroinflammation, oxidative damage, and neuronal cell death, thereby restoring neuro‐glial unit integrity and functionality (**Figure** **8**A). Given that RES‐CTP@GPXP restored the functionality of neuro‐glial unit and conferred robust neuroprotection, we further conducted a transcriptional analysis to preliminarily elucidate the therapeutic mechanism. Herein, RNA sequencing analysis was conducted to assess gene expression regulations and identify differentially expressed genes (DEGs) at the ipsilateral basal cortex on day 3 post‐SAH between the saline‐treated and RES‐CTP@GPXP‐treated groups. Hierarchical clustering analysis based on gene expression profiles revealed a distinct separation between these two treatment groups, indicating substantial transcriptomic divergence induced by the therapeutic intervention (Figure 8B). RNA sequencing analysis identified a total of 1049 significant DEGs between the two groups, defined by a threshold of |log2 fold change(FC)|≥ 1 and *P* < 0.05, as illustrated in the corresponding volcano plot (Figure 8B). Among these, 280 genes exhibited a significant upregulation, while 769 genes were markedly downregulated in the RES‐CTP@GPXP‐treated group compared to the saline‐treated group (Figure 8C). Functional annotation and pathway enrichment of these DEGs were conducted using Gene Ontology (GO), Kyoto Encyclopedia of Genes and Genomes (KEGG), Reactome, and Gene Set Enrichment Analysis (GSEA) (Figure 8D–I).

First, we investigated the therapeutic effects of RES‐CTP@GPXP on mitigating neuroinflammation following SAH. GO functional enrichment analysis indicated that RES‐CTP@GPXP treatment can significantly suppress glial‐driven inflammatory pathways. Key downregulated pathways included IL‐6 production (typical genes Myd88, Tlr1, Tlr4, and Tnf downregulated; *p* = 6.82e‐11), Regulation of IL‐6 biosynthetic process (*p* = 4.27e‐6), Tumor necrosis factor production (typical genes Cybb, Myd88, and Tlr1 downregulated; *p* = 6.35e‐10), Regulation TLR/TLR9 signaling pathway (typical genes Tlr1, Cd36, Pk3ap1 and Rsad2 downregulated; *p* = 3.94e‐6), and Regulation of NF‐κB signaling (typical genes Myd88, Tlr4, and Tnf downregulated; *p* = 2.65e‐5), and Regulation of inflammatory response (*p* = 3.86e‐13; Figure 8D). KEGG enrichment further confirmed downregulation of TLR signaling pathway (typical genes Irlf7, Irf9, Stat1, and Tnf downregulated; *p* = 1.20e‐11), NF‐κB signaling (typical genes Tnf, Tlr4, Ddx58 and Trim 25 downregulated; *p* = 7.56e‐11), and TNF signaling pathway (typical genes Tnf, Ccl12 and Mlkl downregulated; *p* = 1.69e‐12) (Figure 8E,J). Reactome pathway analysis further demonstrated that RES‐CTP@GPXP exhibited anti‐inflammatory characteristics through downregulating relevant inflammatory pathways, i.e., IL‐6 signaling (typical gene Stat1 downregulated; *p* < 0.05), IL‐6 family signaling, TLR cascades (typical genes CD14 and Tlr4 downregulated; *p* < 0.05), TLR9 cascade (typical genes Myd88 and Irf7 downregulated), and Regulation of TLR by endogenous ligand (typical genes Tlr1, CD14, and Tlr4 downregulated; Figure 8F). The GSEA validated the broad inhibition of the TLR signaling pathway (*p <* 0.001) and NF‐κB signaling (*p <* 0.001) (Figure 8H,I). The representative genes from relevant pathways were also identified, such as Stat1, Cd14, Trim25, and Irf7, which were significantly downregulated (Figure 8J). The results from multiple enrichment analysis databases underscored RES‐CTP@GPXP's capacity to modulate glial activation and suppress neuroinflammation.

Furthermore, RES‐CTP@GPXP treatment demonstrated the therapeutic effects against oxidative damages. GO enrichment analysis revealed that RES‐CTP@GPXP treatment resulted in the upregulation of molecular function genes associated with the Antioxidant activity, Glutathione binding, Oxidoreductase complex, as well as upregulating relevant biological process pathways (i.e., Regulation of cellular response to oxidative stress and Response to oxidative stress), and downregulation of ROS metabolic process, suggesting that it enhances neuron‐intrinsic antioxidant defense (Figure S12, Supporting information). KEGG pathway analysis also indicated that the Glutathione metabolism pathway was upregulated (Figure 8E), suggesting that RES‐CTP@GPXP has antioxidant capabilities. These findings underscored the ability of RES‐CTP@GPXP to restore redox homeostasis through enhanced antioxidant defense. Additionally, RES‐CTP@GPXP treatment also significantly suppressed multiple death‐related pathways. GO enrichment analysis showed that RES‐CTP@GPXP treatment could inhibit neuron cell death by downregulating various pathways, i.e., Regulation of neuron death(typical genes Hmox1, Apaf1,Tnf, and Tlr4 downregulated), Regulation of apoptotic signaling pathway (typical genes Hmox1, Apaf1, and Ctsc downregulated), Inflammatory cell apoptotic process, Regulation of neuron apoptotic process (typical genes Hmox1, Ccl12, and Tnf downregulated), Programmed necrotic cell death (typical genes Mlk1 and Tnf downregulated), and Regulation of oxidative stress‐induced cell death (typical genes Tlr4 downregulated) (Figure 8D,J). KEGG enrichment analysis revealed the downregulation of Ferroptosis and Apoptosis pathways (Figure 8E). Moreover, Reactome analysis also showed the downregulation of cell death pathways, such as Regulation of necroptotic cell death and Programmed cell death (Figure 8F). These data indicate that RES‐CTP@GPXP preserves neuronal integrity by mitigating oxidative and inflammatory cytotoxic cascades.

Taken together, convergent evidence from cross‐platform enrichment analyses (GO/KEGG/Reactome/GSEA) revealed that RES‐CTP@GPXP exerts its therapeutic effects by modulating the neuro‐glial unit, a dynamic cellular network critical maintaining neurovascular homeostasis. RES‐CTP@GPXP promotes neuro‐glial unit integrity by: i) attenuating neuroinflammation, ii) mitigating oxidative damage, iii) preventing neuronal cell death. GO enrichment analysis mapped these protective effects to neuro‐glial unit remodeling, with significant downregulation of pathways associated with neuronal loss (e.g., Neuron death and Regulation of neuron death) and aberrant glial cell activation (e.g., Glia cell activation and Microglia cell activation) (Figure 8K). Subsequent protein–protein interaction (PPI) network analysis revealed that genes involved in these pathways form a functionally integrated network (Figure 8L), suggesting extensive molecular crosstalk between neurons and glia. These networks coordinate neuronal survival and glial inflammatory responses,^[^ ^44^, ^45^ ^]^ and their modulation by RES‐CTP@GPXP further supports the therapeutic role of this system in restoring neuro‐glial unit integrity.

Further, several validated experiments were conducted based on the aforementioned bioinformatics analysis results. First, immunohistochemical staining (IHC) result revealed that RES‐CTP@GPXP treatment group had fewer TLR9‐positive cells than saline group at the ipsilateral basal cortex on day 3 post‐SAH (Figure 8M; Figure S13, Supporting information), indicating that RES‐CTP@GPXP could indeed inhibit the TLR9 activation in vivo. Afterward, RT‐PCR assay was conducted to validate the transcriptomic findings by assessing the mRNA expression levels of key transcriptional regulators, including *Rela*, *Tnf*, and *Il6*. As depicted in Figure 8N‐P, the RES‐CTP@GPXP treatment significantly downregulated the expression of these genes compared to saline group, suggesting suppression of NF‐κB signaling activation. Consistently, ELISA analysis revealed reduced pro‐inflammatory cytokine levels, including TNF‐α and IL‐6, in the RES‐CTP@GPXP‐treated group (Figure 8Q,R), further supporting the anti‐inflammatory effect of the nanotherapeutic intervention. These results validated that RES‐CTP@GPXP treatment reduced neuroinflammation by blocking TLR9 and NF‐κB signaling axis.

Collectively, the RNA sequencing analysis and in vivo experiments all revealed significant alterations in the gene expression profiles and signaling pathways associated with the regulation of neuro‐glial units, preliminarily shedding light on the therapeutic mechanism of RES‐CTP@GPXP.

### 2.10 In Vivo Biosafety Assessment

In vivo biosafety is a critical prerequisite for clinical applications of therapeutic agents. To systematically assess the biosafety of RES‐CTP@GPXP, we conducted multi‐dimensional toxicity analyses encompassing hematological, biochemical, and histopathological assessments at 7‐ and 28‐days post‐administration. First, hematological examination and biochemical test were performed on blood samples, revealing no significant abnormalities in hematological parameters or hepatic and renal function biomarkers (Figures S14 and S15, Supporting information). These results suggest that RES‐CTP@GPXP does not adversely affect blood cell counts or liver and kidney functions. Besides, to further evaluate potential systemic toxicity, major organs including brain, heart, liver, spleen, lung, and kidney were collected and subjected to H&E staining. Histopathological examinations revealed no observable tissue damages, inflammatory cells infiltration, and other abnormalities in any organ (Figures S16 and S17, Supporting information). In conclusion, these findings demonstrated that in situ proliferating peptide nanoparticle RES‐CTP@GPXP has ignorable considerations about its short‐ and long‐term toxicity and off‐target effects, thereby reinforcing its safety profile for potential clinical applications.

### 2.11 Further Validation of the Therapeutic Efficacy and Safety in a Rat SAH Model

To enhance the translational robustness of our findings beyond a single species, we established an independent SAH model in rats following the same surgical procedure and treatment regimen as used in mice (Figure S18, Supporting information). Peptide nanoparticles were intranasally administered at 4 mg kg^−1^ across five treatment groups (saline, GPXP, CTP, CTP@GPXP, RES‐CTP@GPXP). Consistent with murine results, RES‐CTP@GPXP significantly promoted neuroanatomical recovery, as evidenced by the improved cortical structure (Figure S19A, Supporting information) and increased numbers of Nissl‐positive neurons (Figure S19B, Supporting information). Moreover, Kaplan–Meier survival analysis revealed a higher 7‐day survival rate in the RES‐CTP@GPXP group (*p* = 0.025, Figure S19C, Supporting information). Besides, safety evaluations, including routine blood tests, serum biochemistry, and histopathological examination of major organs, showed no obvious systemic toxicity or organ damage (Figure S20A–I, Supporting information). These findings support the therapeutic efficacy and safety of RES‐CTP@GPXP in a second rodent model, further demonstrating its translational potential.

### 2.12 Study Limitations

This study explored pathological cfDNA as a potential therapeutic target, revealing its correlation with SAH severity and poor prognosis. Based on these findings, we developed an in situ proliferating peptide nanoparticle (RES‐CTP@GPXP) with a flexible multi‐target therapeutic function to reduce SBI following SAH via modulating neuroinflammation, oxidative damages, and neuronal survival. However, the current study has several limitations. First, multi‐target strategies may have certain intrinsic disadvantages, such as causing side effects due to the unexpected interactions among different components. Fortunately, our in vitro and in vivo results demonstrated satisfactory biosafety at therapeutic doses. Next, although cfDNA was identified as a biomarker for SBI and prognosis, its practical prediction value still requires validation in large‐scale clinical trials. Lastly, the use of small animal models inherently limits the generalizability of the results to humans. Although our findings were validated in mice and rat SAH models, further investigation in large‐animal models or patient‐derived organoid systems is warranted.

## 3 Conclusion

As proof of our hypothesis, this work revealed a significant correlation between elevated serum cfDNA levels, severity of brain injury, and a 3‐month poor prognosis in aSAH patients. Then, we constructed the in situ proliferating peptide nanoparticle (RES‐CTP@GPXP) for the flexible multi‐target therapy of SBI following SAH. RES‐CTP@GPXP could split into the individual CTP and GPXP under pathological MMP‐2 action, where CTP could scavenge pathological cfDNA, inhibit abnormal activation of TLR9 and downstream NF‐κB signaling in microglia, and the GPXP preferentially targeted to neurons for inhibiting oxidative damage and neuronal cell death. Further, animal experiments proved its therapeutic effects on alleviating neuroinflammation and oxidative stress, inhibiting neuronal apoptosis and ferroptosis, ultimately effectively reducing SBI and promoting neurofunctional recovery, which was superior to the single‐target therapy and the simply‐combining dual‐target therapy. Further, RES‐CTP@GPXP was also proved with the satisfactory biosafety in a therapeutic dose. This work not only underscored the pivotal role of cfDNA in SAH but also proposed a novel “in situ proliferation” concept for the design principle of multi‐target drugs.

## 4 Experimental Section

Materials and experiment details can be seen in Supporting Information.

### Statistical Analysis

Statistical analyses were performed with GraphPad Prism (version 10.0, California, USA) and MedCalc software (version 20.0.4, Ostend, Belgium). Multiple groups were tested via one‐way ANOVA, and comparisons between two groups were performed using Unpaired Student's *t*‐test (two‐tailed). To account for potential confounders, weighted univariate and multivariable analyses were conducted. Factors that showed statistical significance (*p* < 0.05) in the weighted univariate analysis were subsequently included in the weighted multivariable logistic regression model. ROC and AUC of ROC were performed to evaluate the performance of serum cfDNA in predicting brain injury for aSAH patients. Survival curves were generated utilizing the Kaplan‐Meier approach and subsequently analyzed with a log‐rank test to identify differences in survival rates. Data are expressed as mean ± SEM and *p* < 0.05 was considered statistically significant. *p*‐values are indicated as ^*^ *p* < 0.05, ^**^ *p* < 0.01, ^***^ *p* < 0.001, and ^****^ *p* < 0.0001.

### Ethical Approval Statement

The animal experiments followed the Animal Protection Guidelines of Fujian Medical University (IACUC FJMU 2022‐0608) and also aligned with the “Guide for the Protection and Use of Experimental Animals” of the American National Institutes of Health.

The study protocol for human participants received approval from the Ethics Committee of the First Affiliated Hospital of Fujian Medical University (Approval No.: MRCTA, ECFAH of FMU [2022]307 and MRCTA, ECFAH of FMU [2022]601). Written informed consent was obtained from patients or patient families or legal guardians.

## Conflict of Interest

The authors declare no conflict of interest.

## Author Contributions

Y.B.Z., P.S.Y., and F.X.C. contributed equally to this work. D.Z.K. and B.G. proposed and supervised the project. B.G., Y.B.Z., P.S.Y., F.X.C., X.G.N., and H.J.W performed physicochemical characterizations. Y.B.Z., P.S.Y., and F.X.C. performed in vitro and in vivo intervention effects and biosafety. Y.B.Z. performed intervention mechanism experiments. H.J.W and S.F.Z collected clinical information and blood samples. D.Z.K., B.G., Y.B.Z. and P.S.Y. contributed to discussions about experimental results. Y.B.Z., B.G., and P.S.Y. wrote this manuscript and supplementary materials. All authors have given approval to the final version of the manuscript.

## Supporting information



Supporting Information

## Data Availability

The data that support the findings of this study are available in the supplementary material of this article.
