# Supplementary material for: In Situ Proliferating Peptide Nanoparticle Augments Multi‐Target Intervention of Secondary Brain Damage Following Subarachnoid Hemorrhage
Source: Adv Sci (Weinh). 2025 May 30;12(31):e17456. doi: 10.1002/advs.202417456 (PMC12376667; doi:10.1002/advs.202417456)
Supplement: Supplementary file 1 — Supporting Information [file ADVS-12-e17456-s001.docx]

**Supplementary Information for**

**In Situ Proliferating Peptide Nanoparticle Augments Multi-Target Intervention of Secondary Brain Damage Following Subarachnoid Hemorrhage**

*Yibin Zhang, Peisen Yao, Fuxiang Chen, Shufa Zheng, Xuegang Niu, Haojie Wang, Yuanxiang Lin, Bin Gao^*^, and Dezhi Kang^*^*

**Author Information**

Y. Zhang, P. Yao, F. Chen, S. Zheng, X. Niu, H. Wang, Y. Lin, B. Gao, D. Kang

Department of Neurosurgery, Neurosurgery Research Institute, The First Affiliated Hospital, Fujian Medical University, Fuzhou 350005, Fujian, China.

Y. Zhang, P. Yao, F. Chen, S. Zheng, X. Niu, H. Wang, Y. Lin, B. Gao, D. Kang

Department of Neurosurgery, National Regional Medical Center, Binhai Campus of the First Affiliated Hospital, Fujian Medical University, Fuzhou 350212, China.

Y. Zhang, P. Yao, F. Chen, S. Zheng, X. Niu, H. Wang, Y. Lin, B. Gao, D. Kang

Fujian Provincial Institutes of Brain Disorders and Brain Sciences, The First Affiliated Hospital, Fujian Medical University, Fuzhou 350005, Fujian, China.

^*^Corresponding author: Bin Gao, gaobin11@tju.edu.cn; Dezhi Kang, kangdezhi@fjmu.edu.cn.

**Experimental Section**

**Materials**

Peptides were purchased from Heifei KS-V Peptide Biotechonology CO., LTD (Hefei, China). 2′,7′-dichlorofluorescein diacetate (DCFH-DA) assay kit, TUNEL apoptosis assay kit and 4′, 6-diamidino-2-phenylindole dihydrochloride (DAPI) were obtained from Meilun Biotechnology Co., Ltd. (Dalian, China). Dulbecco’s modified eagle medium (DMEM), fetal bovine serum (FBS), cell counting kit-8 (CCK-8) and Quant-iT Picogreen dsDNA assay kit was ordered from Invitrogen Biotechnology Co., Ltd (CA, USA). DPPH free radical scavenging capacity assay kit, IL-6 ELISA kit, and TNF-α ELISA kit were obtained from Solarbio Science & Technology Co., Ltd. (Beijing, China). ABTS assay kits were purchased from Beyotime Institue of Biotechonology (Haimen, China). Hyaluronidase was purchased from Sigma-Aldrich(Shanghai,China). Primary antibodies for immunofluorescence and immunohistochemistry experiments, including MMP-2 (ab181286), TLR9 (ab134368), GPX4(ab125066), GFAP (ab7260), Iba-1 (Cat#019-19741), 4-HNE (ab48506), MDA (ab27642), 8-OHG (ab48508) and NeuN (ab177487) were purchased as indicated. Other chemicals were analytical grade without further purification.

***Formulation of RES-CTP@GPXP:*** Lyophilized RES-CTP and GPXP peptide powders were dissolved in PBS (pH = 7.4) at a concentration of 5 mM and then were incubated for 30 min at 25 °C, resulting in the formation of RES-CTP@GPXP.

***Antioxidant potential assessment:*** The antioxidant potential was evaluated through DPPH and ABTS assays. In brief, ethanol solution of DPPH was mixed with varying concentrations of samples. The mixtures were then incubated in a shaking water bath at approximately 37 °C and 100 rpm for 30 min to ensure adequate reaction time. Finally, the absorbance value at 515 nm was measured to reflect antioxidant potential, where the pure DPPH solution was used as a control. Similarly, ABTS assay was also performed by ABTS kit according to the manufacturer’s protocol and quantified by multifunctional microplate reader.

***Cell culture and establishment of a cell model of SAH:*** The BV2, HT22 cell lines were obtained from the Cell Bank of Typical Culture Collection of the Chinese Academy of Sciences and maintained in a 5 % CO_2_ incubator at 37 °C. Cells were seeded in a 96-well plate and cultured for 24 h with complete DMEM, and then hemin was added for 6 hours of incubation. Next, the culture medium was replaced by a fresh medium containing CCK-8 agent, and incubation continued for an additional 2 h. Cell viability was then assessed by measuring absorbance at 450 nm using a microplate reader, enabling the identification of the optimal hemin concentration for subsequent experiments.

***In vitro evaluation of ROS regulation:*** The DCFH-DA assay kit was employed *in vitro* to assess intracellular ROS levels. Cell model was established by treating HT22 cells with hemin at a concentration of 60 μM. Then, the cells were incubated with various drugs for 24 h, followed by a 30-min exposure to DCFH-DA. After three washes with PBS to eliminate unreacted dye, cells were trypsinized and resuspended in PBS. Fluorescence microscopy was employed to visualize intracellular ROS, and mean fluorescence intensity (MFI) was quantified using ImageJ software.

***In vitro evaluation of DNA-binding capability:*** DNA-binding capability was evaluated using Quant-iT PicoGreen dsDNA assay. Calf thymus DNA was mixed with the PicoGreen reagent in Tris-EDTA buffer and incubated in dark for 10 minutes to ensure optimal fluorescence conditions. Then, RES-CTP@GPXP was pre-incubated for 24 h in the presence or absence of MMP-2 before being introduced to calf thymus DNA solution for an additional 30-minute incubation in dark. Fluorescence intensity was subsequently measured at 520 nm to quantify DNA binding affinity.

***In vitro* evaluations of neuroprotective effect and cytotoxicity:** HT22 cell lines were treated with hemin (60 μM) to establish a cell model of SAH. Cells were cultured for 24 h with varying concentrations of drugs, after which culture medium was replaced with a fresh medium containing the CCK-8 reagent for an additional 2 h incubation. Absorbance was measured at 450 nm using a microplate reader to assess cell viability, and thereby evaluating the neuroprotective effects *in vitro*. Additionally, cytotoxicity was evaluated using the same protocol in untreated HT22 cells to serve as a control.

***Clinical sample collection:*** Forty-five adult patients with aSAH had SAH symptom onset (e.g. sudden-onset thunderclap headaches or loss of consciousness) within 6 h. Clinical parameters were prospectively collected, including Hunt & Hess grade and mFisher scale. Peripheral venous samples were collected from patients within 2 h of hospital admission after written informed consent. Excluded criteria included a prior history of strokes, infectious or inflammatory diseases, malignancies, immunosuppressive therapy, and hepatic, renal, or cardiac dysfunction. A control group comprised 30 age-matched healthy individuals who underwent a physical examination. The characteristics of healthy controls and SAH patients were present in Table S1, S2. No significant differences were observed in the age and gender between the two groups. The blood collection time from onset of aSAH was 7.0(4.0-12.50) h. All blood samples were centrifuged, and the supernatant serum was stored at −80 °C for future analyses.

***TLR9 activation:*** To study TLR9 activation in HEK-TLR9 reporter cells,PBS, CpG (0.5 μM), and peptide nanoparticles were incubated with HEK-TLR9 reporter cells in a 96-well plate for 24 h. Then, the supernatants were collected, and QUANTI-Blue assay was performed according to the manual instructions. The secreted embryonic alkaline phosphatase (SEAP) activity was quantified by the optical density at 620 nm (OD_620_). Results were presented as the percentage absorbance of cells that were treated with CpG. Additionally, the TLR9 activation in HEK-TLR9 reporter cells by the healthy human serum and aSAH patients’ serum were also studied according to the above experimental process.

***Establishment of SAH model and therapy strategy:*** C57BL/6 mice (male, weighing 22-27g) were used and were purchased from Silake Experimental Animal Limited Liability Company (Shanghai, China). Mice were housed under constant temperature and humidity conditions with a 12-hour day/high cycle and a free access to food and water. Mice were randomly divided into six groups (sham, saline, GPXP, CTP, CTP@GPXP, and RES-CTP@GPXP) using a simple randomization of excel-generate random numbers. SAH model was performed for the later five groups. The endovascular perforation technique was used to establish the mouse model of SAH ^[1]^. Briefly, mice were initially anesthetized by inhalation of 2% isoflurane supplemented with O_2_ during surgery. After exposing the left carotid artery, the external and internal carotid arteries were carefully separated. The left external carotid artery (ECA) was isolated, and a blunt 5-0 nylon suture was advanced through the ECA into the internal carotid artery until resistance was encountered at the bifurcation of the anterior and middle cerebral arteries. The suture was advanced further to puncture the vessel, and then it was immediately withdrawn to induce hemorrhage. The sham mice underwent the surgical procedures without vessel puncture. Throughout the procedure, vital signs were under continuous observation, including respiration rate, heart rate, skin pigmentation, and pedal reflex (firm toe pinch). To ensure that the animal(s) were not in distress and were responding appropriately to the anesthesia and procedure, we monitored these vitals every 5 min. The mice were kept on a heating pad to maintain body temperature during the surgery and recovery. All surgical procedures were conducted by a single experienced investigator who was blinded to the experimental subgroups.

For drug administration, the mice of each group (Saline, GPXP, CTP, CTP@GPXP, RES-CTP@GPXP) were intranasally administrated at 4 mg/kg at 1 h after surgery as previously described ^[2, 3]^. Briefly, mice were anesthetized with 2% isoflurane and positioned in a supine posture to facilitate intranasal administration. To enhance drug absorption across the nasal mucosa, hyaluronidase was pre-administered at a dose of 50 U in 5 µL per nostril (total of 100 U per mouse). This pretreatment was conducted 30 minutes prior to the intranasal delivery of the therapeutic agents, thereby promoting mucosal permeability and ensuring efficient drug uptake^[3]^. A total volume of 10 μL of the drug was administered, alternating 2 μL in one naris every 5 minutes. Mice were laid on their dorsal side for a duration exceeding 30 minutes to aid in the absorption of the drugs through the olfactory and trigeminal nerve pathways. The sham group was performed with the same protocol but without drug administration.

Representative images of brains from mice in sham and SAH groups are shown in **Figure S21A-B.** We specifically focused on the ipsilateral basal cortex (Figure 5D).

To enhance the translational robustness of our findings beyond a single species, we established an independent SAH model in rats following the same surgical procedure and treatment regimen as used in mice (Figure S18-S20). The male Sprague Dawley (SD) rats (weighing 250-300 g) were used and were purchased from Silake Experimental Animal Limited Liability Company (Shanghai, China). A blunt 2-0 nylon suture was employed during operation. Peptide nanoparticles were intranasally administered at 4 mg/kg (volume: 100 μL) 1 h post-SAH across five treatment groups (saline, GPXP, CTP, CTP@GPXP, RES-CTP@GPXP).

***SAH Grade****:* The SAH severity was graded 24 h post-surgery using a standardized scoring system adapted from previous studies ^[4]^. The basal cistern was divided into 6 regions, each assigned a score from 0 to 3 based on the amount of hemorrhage, yielding a total possible score of 0 to 18 (Figure S21C). Mice with a SAH grade ≤ 7 were excluded from the study, as mild SAH did not result in significant neurological impairments, according to prior study ^[5]^. This exclusion criterion ensured that only animals with moderate to severe SAH, which more closely mimics human pathology (aSAH), were included in the final analysis. The SAH grade was calculated to ensure the uniformity and stability of the experimental model. No significant differences were observed in the SAH grade among the different groups 24 h post-SAH (Figure S21D).

***In vivo and ex vivo evaluations of biodistribution:*** Cy7-labeled RES-CTP@GPXP was intranasally administered to the SAH mice at 4 mg/kg. At different time points, the IVIS fluorescence imaging system visualized the mice. Besides, mice were sacrificed to collect brains and other major organs (heart, liver, spleen, lung, kidney, brain, stomach) for *ex vivo* fluorescence imaging.

***In vivo inhibitory effects on neuroinflammation and oxidative damage******:*** To evaluate the inhibitory effects on neuroinflammation *in vivo*, the Quant-iT PicoGreen dsDNA kit, immunofluorescence staining, RT-PCR and ELISA tests were employed. Blood samples were collected from inner canthus 24 h post-SAH to quantify cfDNA levels in serum using the Quant-iT PicoGreen dsDNA kit, according to the manufacturer's protocols.

For immunofluorescence staining and immunohistochemical staining (IHC), mice were anesthetized and transcardially perfused with saline, followed by 4 % paraformaldehyde (PFA) fixation. The brains were harvested and processed into sections approximately 25 μm thick. Following permeabilization with Triton X-100, sections underwent serum treatment to facilitate overnight incubation at 4 °C with primary antibodies, including anti-TLR9, anti-GFAP, anti-Iba-1, anti-8-OHG, and anti-NeuN. After thoroughly washing with PBS, sections were incubated with Alexa Fluor-conjugated secondary antibodies, with DAPI as a nuclear counterstain. For IHC, brain sections were stained with antibodies against TLR9 and GPX4 according to the manufacturer’s protocols.

RT-PCR was conducted on brain tissue samples obtained from the ipsilateral basal cortex, measuring *Rela*, *Tnf*, and *Il6* expression levels. ELISA assays were also performed on both brain tissues and blood samples to measure levels of IL-6 and TNF-α, following the respective ELISA kit instructions.

***In vivo evaluation of inhibiting programmed cell death:*** Following permeabilization with Triton X-100, the sections were treated with serum overnight at 4 °C to facilitate incubation with primary antibodies, including anti-MDA, anti-4-HNE, and anti-NeuN. Following a thorough wash with PBS, the sections were incubated with Alexa Fluor-conjugated secondary antibodies, with DAPI used as a nuclear counterstain. For ELISA assay, the brain tissues at ipsilateral basal cortex were collected to support GSH assay kit and Iron assay kit tests according to their instruments for examining GSH and iron contents. For the TEM observation, mice were perfused with pre-cooled PBS and their brains were promptly removed and fixed in 2.5 % glutaraldehyde. The ipsilateral basal cortex was removed, and then fixed in 2.5 % glutaraldehyde overnight at 4 °C. It was postfixed in 1 % OsO_4_ for 2 h, washed three times in cacodylate buffer, stained in 1 % uranylacetate for 1 h, then dehydrated in the grading concentrations of ethanol, and finally embedded in Epon. Thin tissue sections were sliced using an ultramicrotome from Leica, then mounted on EM grids, stained with uranium acetate and lead citrate, and finally observed under TEM. Moreover, the immunofluorescence staining of NeuN/TUNEL staining was applied to characterize neuronal apoptosis.

***In vivo evaluation of improving neurobehavioral functions:*** The assessment of neurobehavioral functions involved the mGS, open field test, and Y-maze test. mGS assessed the sensorimotor neurological function, including spontaneous activity, movement symmetry of the four limbs, forelimb outstretching, climbing, body proprioception, and response to vibrissae touch. The mGS ranged from 3 to 18, with a higher score indicating better neurological performance and a lower score indicating a more severe neurological deficit. Preoperative mGS were conducted, followed by postoperative mGS on days 1, 2, 3, 5, and 7 after SAH modeling. The results of the last test before SAH modeling were recorded as a baseline. The mice with scores exceeding 13 were excluded on day 1 post-SAH, as this indicated minimal brain injury. All evaluations were conducted daily by the same blinded experimenter to reduce bias.

The open field test assessed locomotor activity. Mice were placed in a 50 cm × 50 cm × 40 cm opaque acrylic arena and allowed to explore for 10 minutes. The arena’s center was marked by a 20 cm × 20 cm square. Movements were recorded using an overhead camera and analyzed with ANY-maze software. Mice were positioned in the corner of the open-field arena. After completing each experiment, the arena was cleaned with 75 % ethanol to remove odors and excrement, ensuring consistency across trials. When the ethanol had evaporated, the subsequent experiments were carried out. The ANY-maze software utilized total traveled distance and average velocity as indicators of locomotor activity. Total distance traveled and average velocity served as indicators of locomotor activity, while anxiety-like behavior was measured by the percentage of time spent in the central zone. Less time spent in the center zone or fewer entries into the inner area indicated stronger anxiety behavior.

The Y-maze test evaluated spatial working memory. The Y-maze, made of white opaque acrylic, consisted of three identical arms (30 cm in length, 5 cm in width, and 15 cm in height) arranged at 120° angles. Mice were placed in one designated arm without prior exposure and allowed to explore for 5 minutes. Their preference for exploring unfamiliar surroundings led them to consistently select an unexplored arm over the recently visited one, which resulted in alternating arm choices during successive trials. Alternations or triads were described as entering three consecutive arms in succession. Then, the percent of alternation (%) was determined by the ratio of the number of triads to the total number of possible alternations. Mice were video-tracked, and their center point needed to pass through the entry zone for a triad to be counted. The apparatus was rotated randomly to prevent any arm preference. ANY-maze software was used to measure spontaneous alternation rate.

***In vivo biosafety assessment:*** *In vivo*, biosafety was evaluated at blood and organ levels, respectively. Blood samples were extracted from the inner canthus on day 7 and 28 for blood routine and blood biochemical tests to reveal physiological index, liver, and kidney functions, which evaluated biosafety at the blood level. Besides, primary organs, including the brain, heart, liver, spleen, lung, and kidney, were collected for H&E staining on day 7 and 28 to detect injury situations, which was used to evaluate biosafety at the organ level.


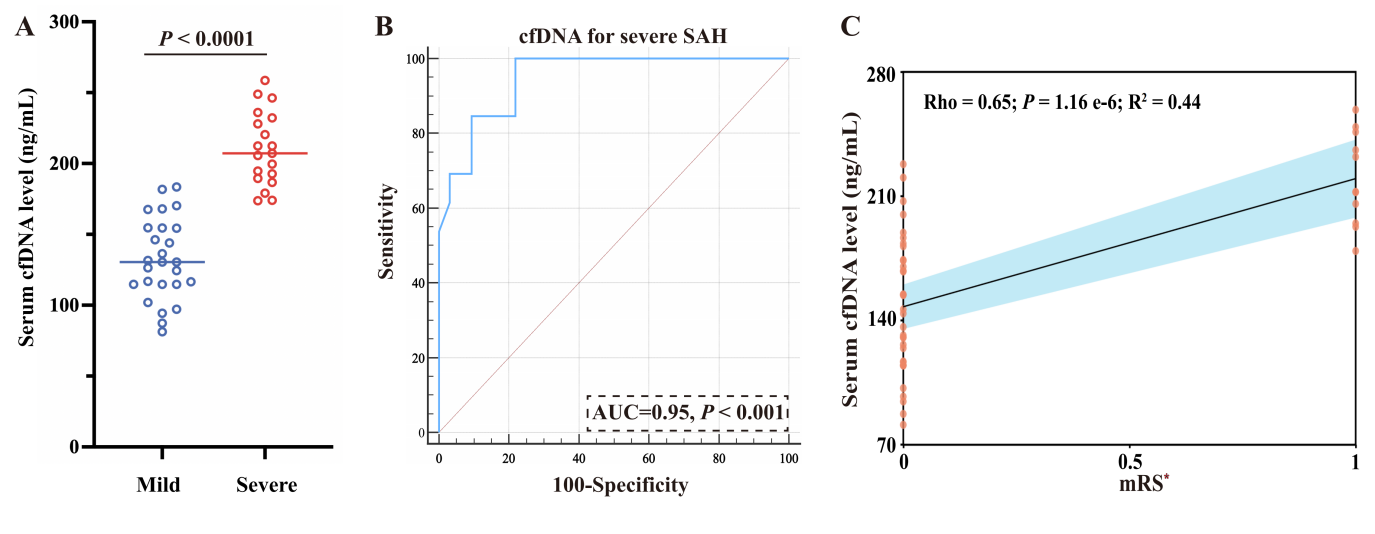


**Figure S1.** Association of serum cfDNA with clinical status and prognosis. A) Comparison of serum cfDNA levels in aSAH patients between mild (Hunt-Hess grade I-II, n = 26) and severe clinical conditions (Hunt-Hess grade III-V, n = 19). B) Receiver operating characteristic curve (ROC) analysis of serum cfDNA levels to predict severe SAH (n = 45). C) Scatter plot of serum cfDNA levels and poor outcomes at 3 months post-discharge(n = 45). The color of the dot represents the group of subjects. The black line is the fitted regression line, and the blue shading is the 95% confidence interval (CI). *mRS represents mRS 3-6, indicating poor outcomes. Data were expressed as mean ± SEM. Statistical comparisons between two groups were performed using unpaired two-tailed Student’s t test.


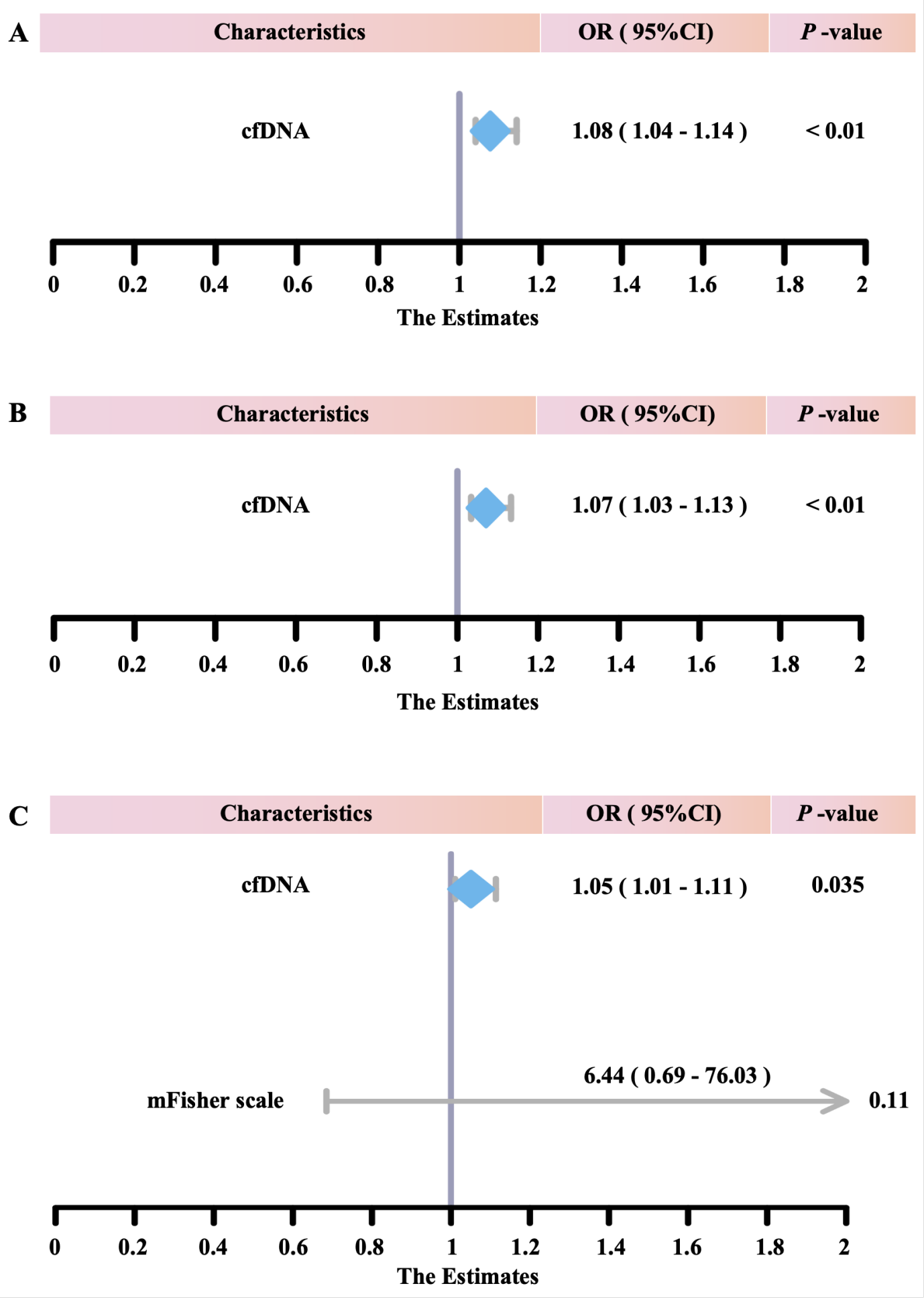


**Figure S2.** Forest plot of univariate/multivariate logistic regression analysis with independent predictors. A) cfDNA predicting severe clinical conditions upon admission (n = 45). B) cfDNA predicting severe SAH upon admission (n = 45). C) cfDNA predicting for poor outcomes 3 months after discharge (n = 45). OR, odds ratio; CI, confidence interval.


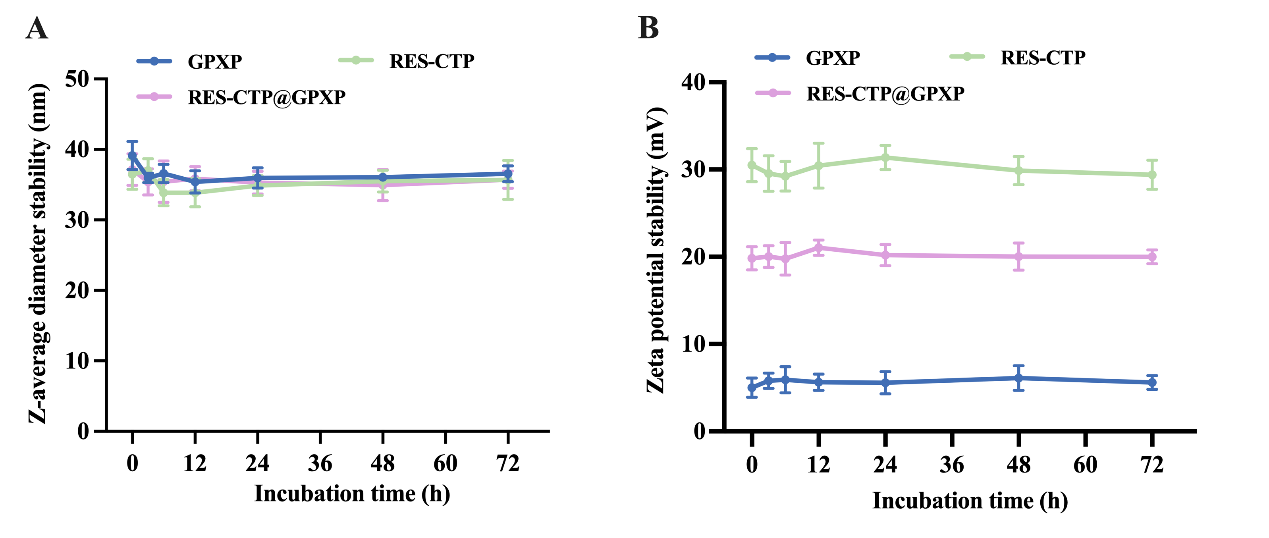


**Figure S3.** Formulation stability at different incubation times. A) Z-average diameter stability (n = 3); B) Zeta potential stability (n = 3). Data were expressed as mean ± SEM.


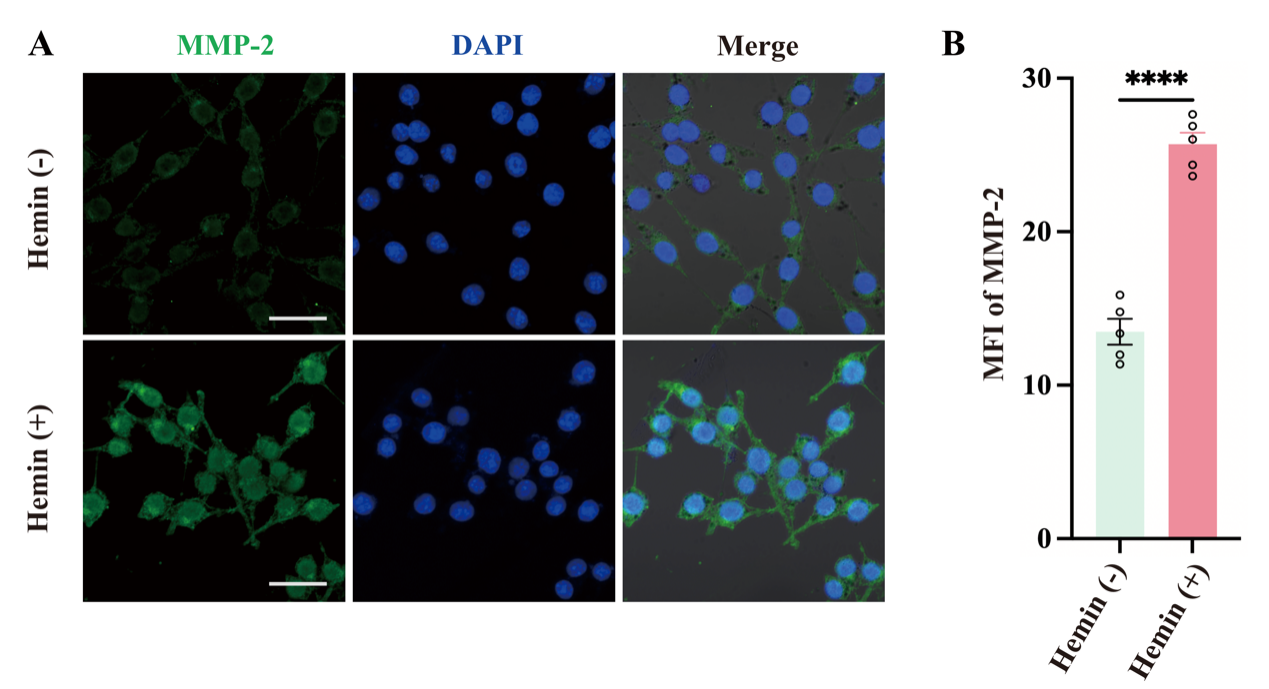


**Figure S4.** A, B) Representative immunofluorescence staining of MMP-2 (green)/ DAPI (blue) in microglia and quantitative analysis of MFI of MMP-2 (n = 5). Scale bars: 20 μm.Data were expressed as mean ± SEM. Statistical comparisons between two groups were performed using unpaired two-tailed Student’s t test. ^****^*p* < 0.0001.


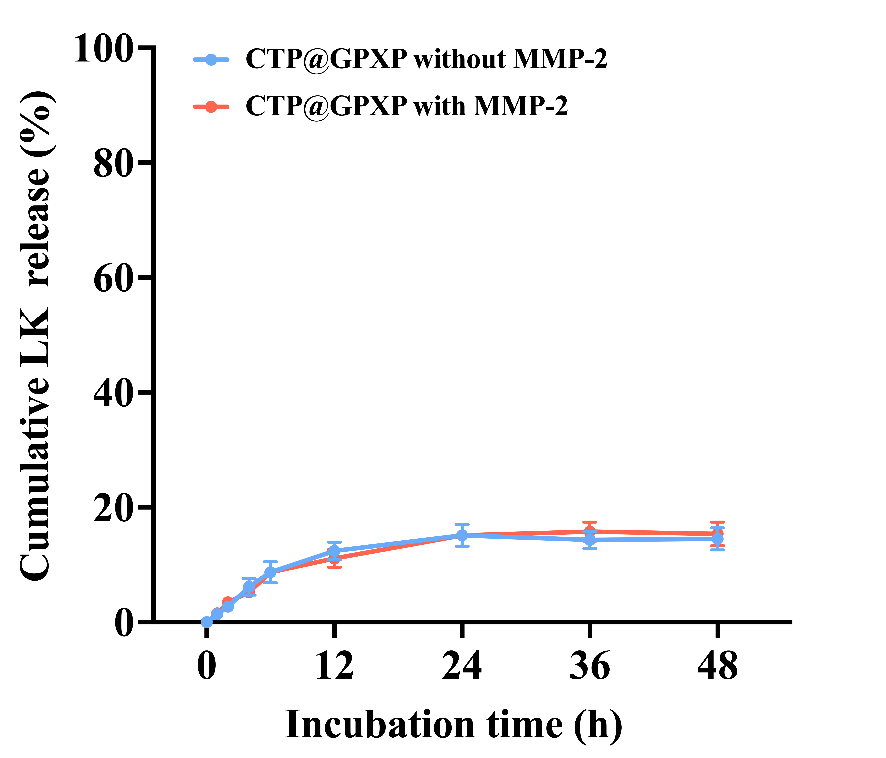


**Figure S5.** Cumulative LK peptide release profile of CTP@GPXP with MMP-2 or without MMP-2 (n = 3). Data were expressed as mean ± SEM.


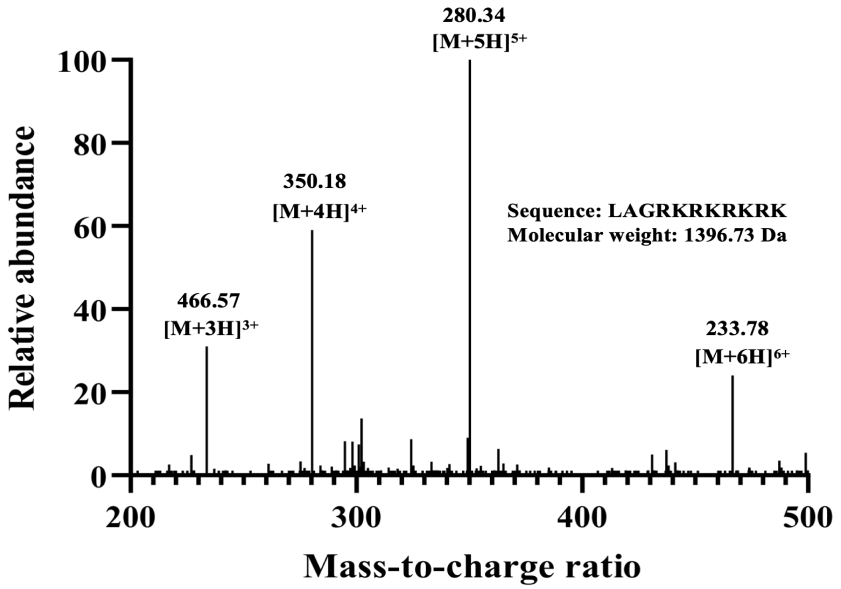


**Figure S6.** ESI-MS spectrum of the released LK peptide after MMP-2 incubation.


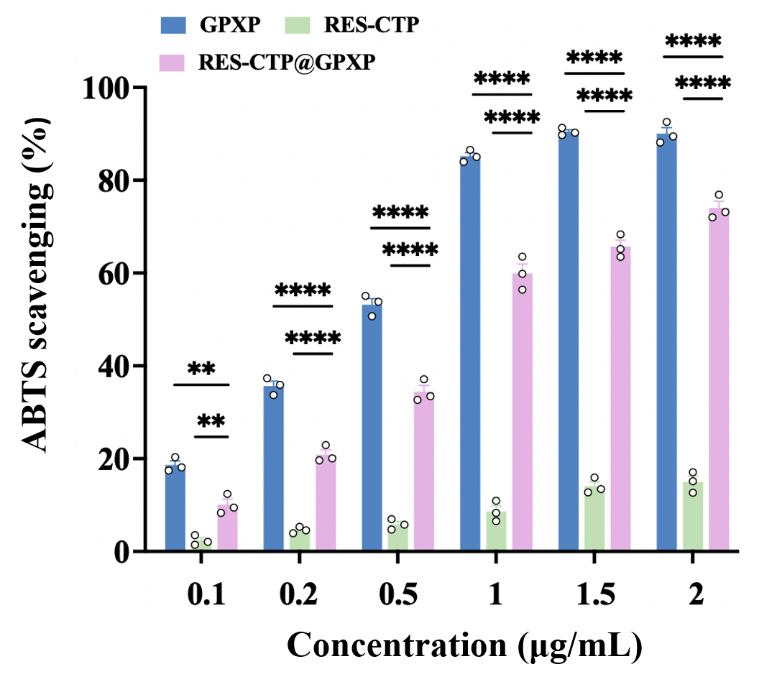


**Figure S7.** ABTS scavenging efficiencies to assess total antioxidant properties (n = 3). Data were expressed as mean ± SEM. One-way ANOVA analysis was used for multiple groups comparison. ^**^*p* < 0.005, ^****^*p* < 0.0001.


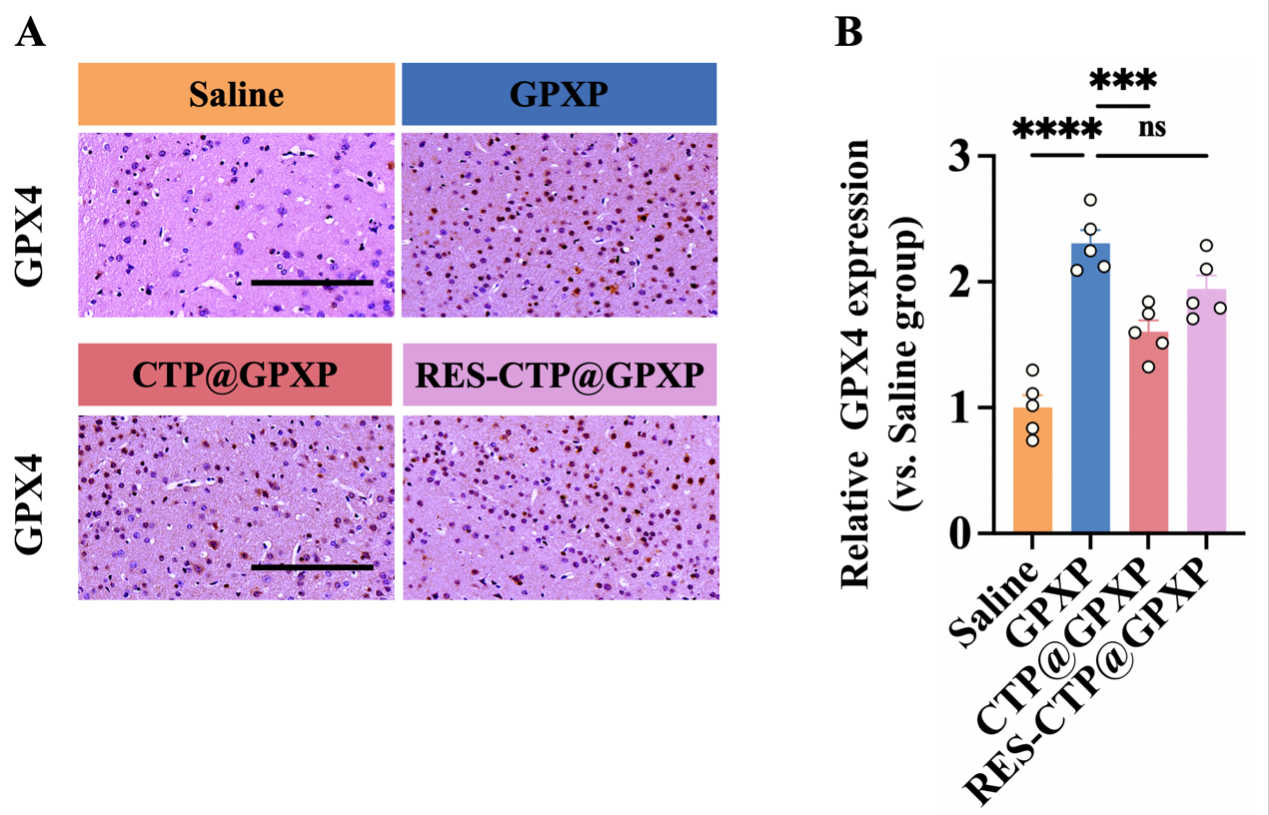


**Figure S8.** A,B) Representative image of GPX4 immunohistochemical staining and quantitative analysis of relative GPX4 expression from the ipsilateral basal cortex on day 3 post-SAH (n = 5). Scale bars: 50 μm. Data were expressed as mean ± SEM. One-way ANOVA analysis was used for multiple groups comparison. ns represents not significant, *p* > 0.05; ^***^*p* < 0.001, ^****^*p* < 0.0001.


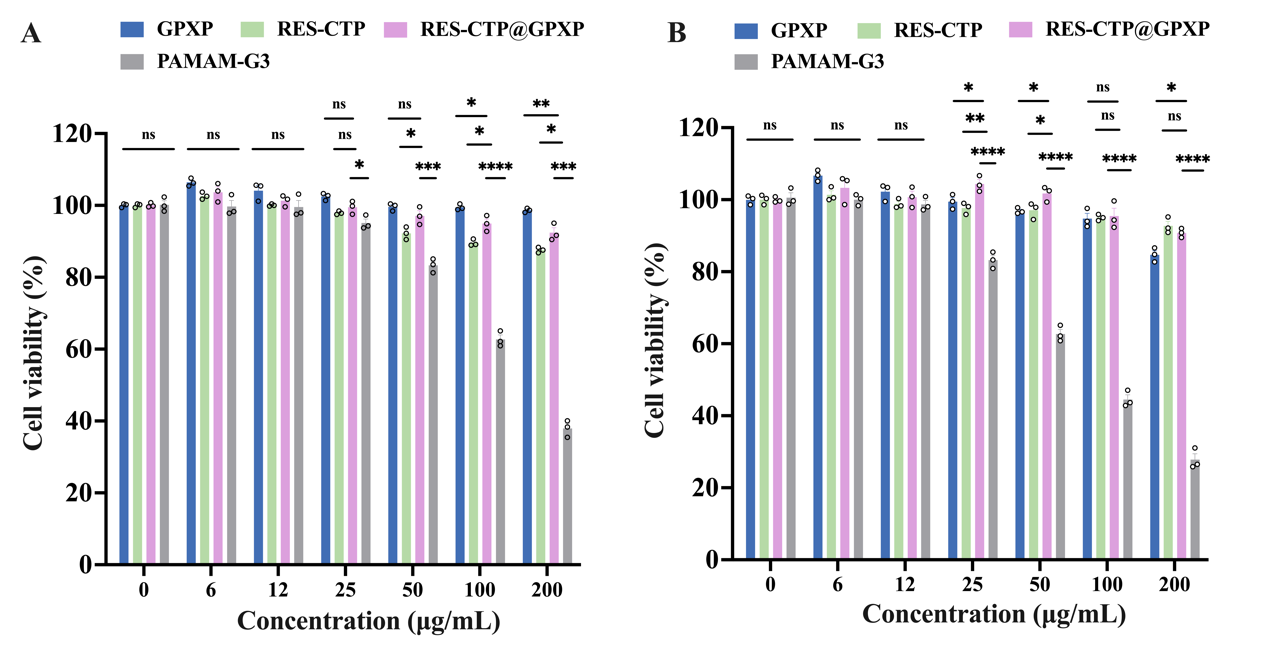


**Figure S9.** Cytotoxicity of different peptide nanoparticles to HT22 and BV2 cells. A) HT22 (n = 3); B) BV2 (n = 3). Data were expressed as mean ± SEM. One-way ANOVA analysis was used for multiple groups comparison. ns represents not significant, *p* > 0.05; ^*^*p* < 0.05, ^**^*p* < 0.005, ^***^*p* < 0.001, ^****^*p* < 0.0001.

**
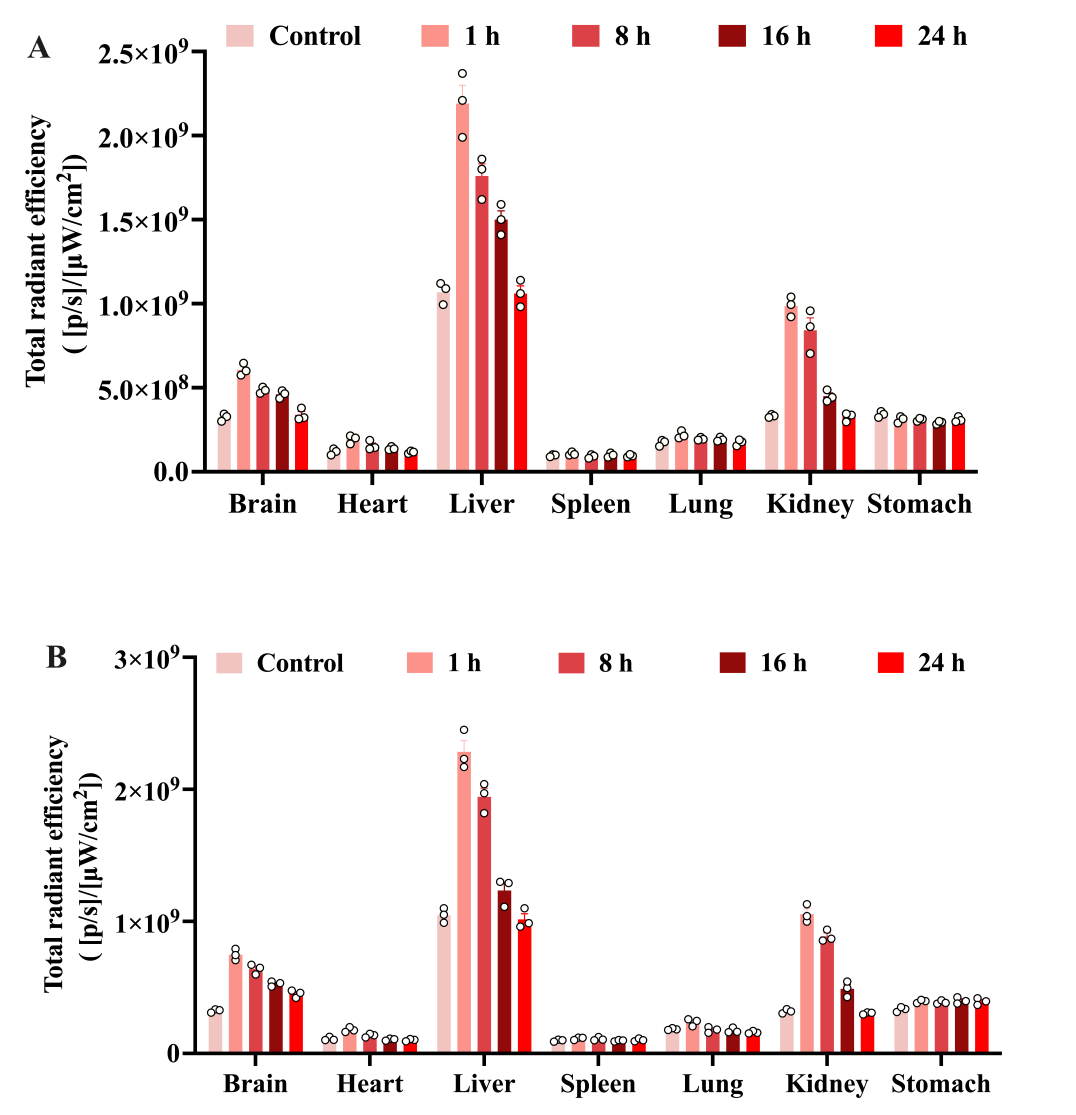
**

**Figure S10.** Intranasal delivery of different drugs following SAH. A) Quantitative analysis of total radiant efficiency *in* *ex vivo* in isolated brains and other major organs following intranasal delivery of GPXP (n = 3). B) Quantitative analysis of total radiant efficiency *in ex vivo* in isolated brains and other major organs following intranasal delivery of CTP@GPXP (n = 3). Data were expressed as mean ± SEM.

**Figure S11.** Representative track plots and heatmap of Y maze test on day 7 following SAH.

**
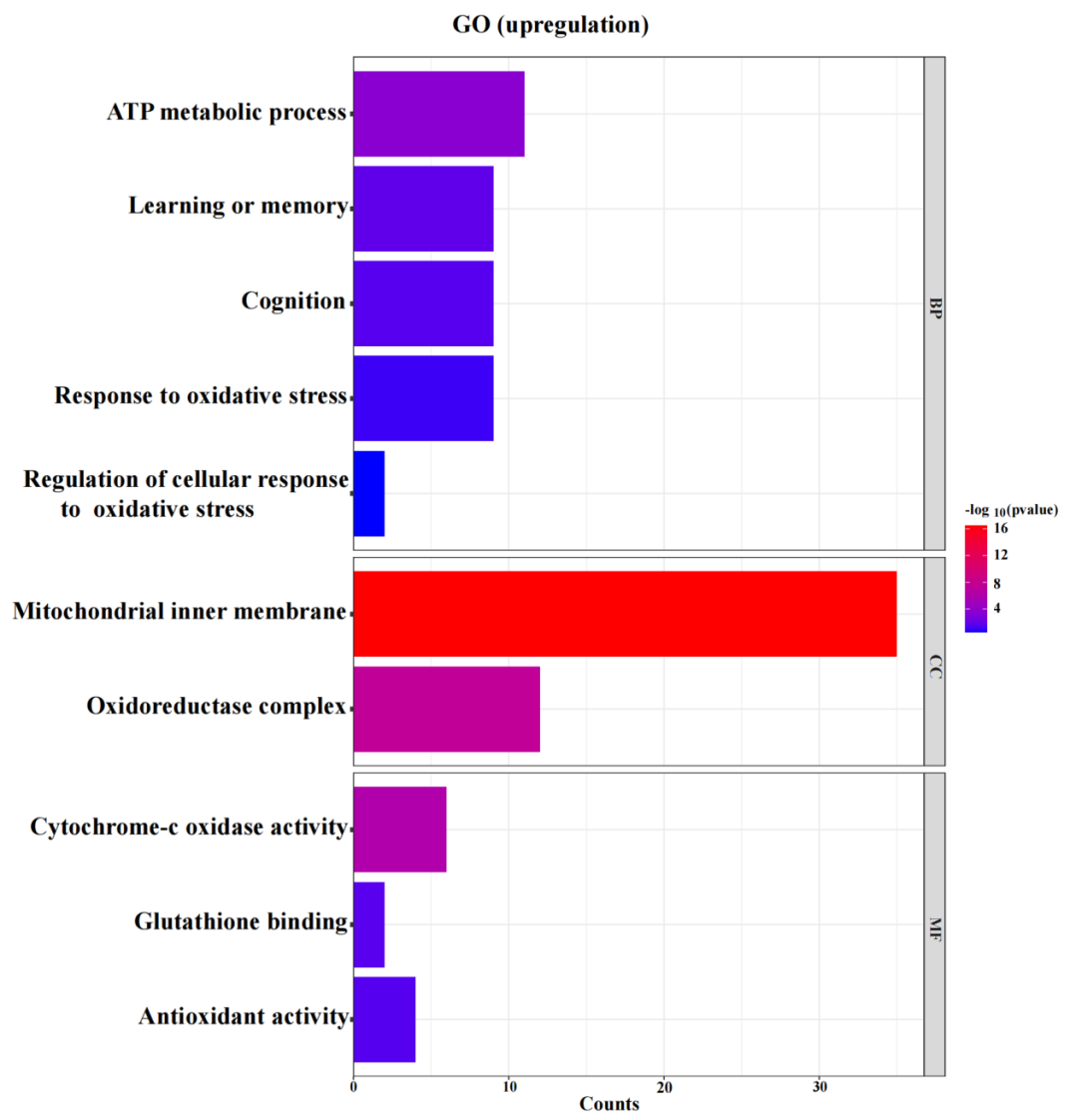
**

**Figure S12.** Enrichment analysis of GO term between RES-CTP@GPXP and saline groups.


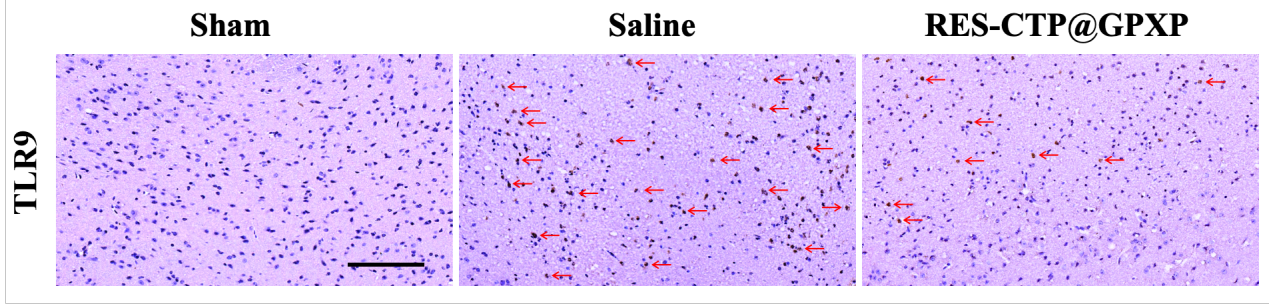


**Figure S13.** Representative image of TLR9 immunohistochemical staining from the ipsilateral basal cortex on day 3 post-SAH. The red arrows show the TLR9 positive cells. Scale bars: 100 μm.

**
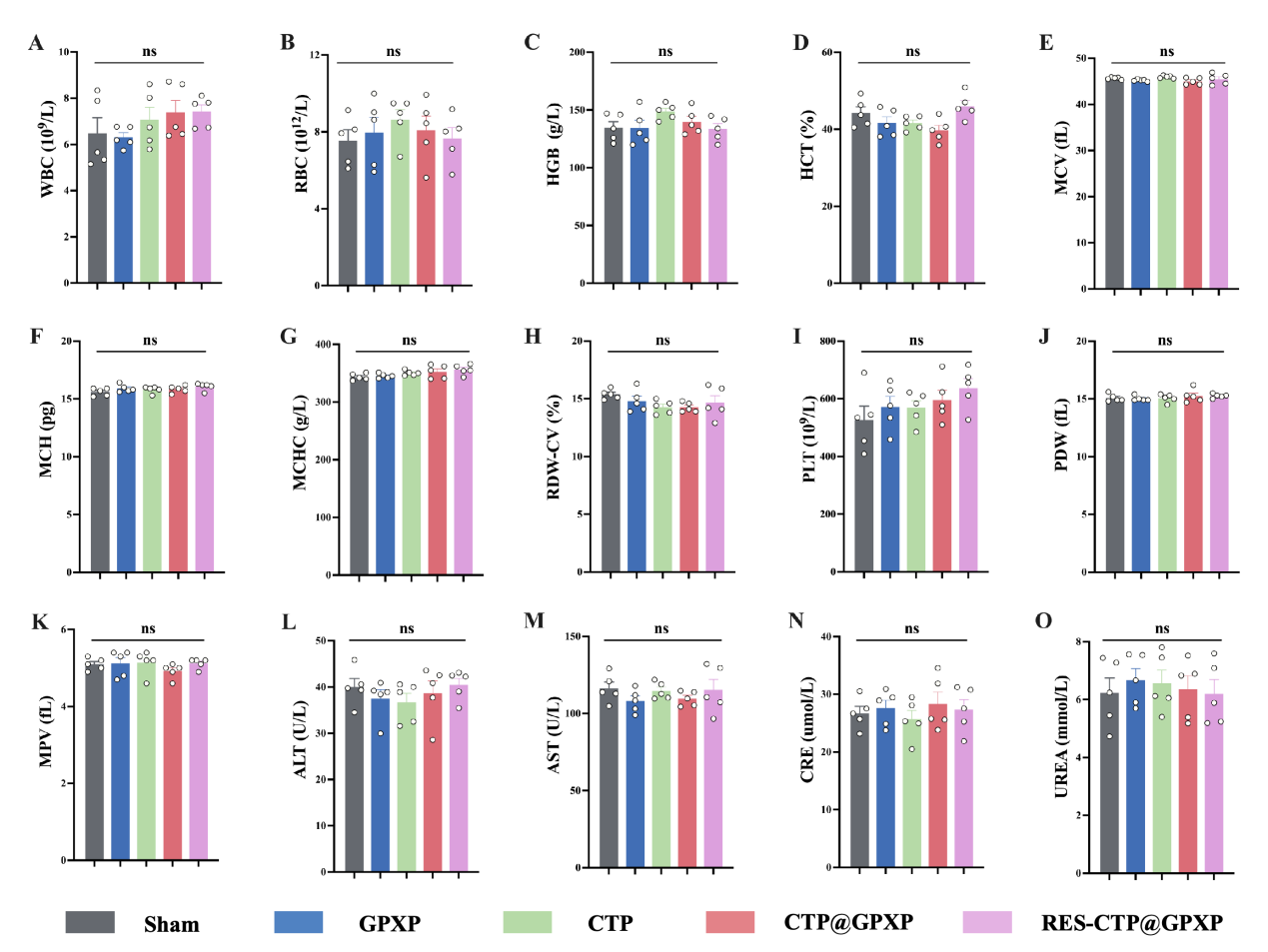
**

**Figure S14.** *In vivo* biosafety assessment at blood level following 7 day of drug administration in mice. (A-K) Side effects on blood cells examined by the blood routine in different groups (n = 5), including A) white blood cell (WBC), B) red blood cell (RBC), C) hemoglobin (HGB), D)hematocrit (HCT), E) mean corpuscular volume (MCV), F) mean corpuscular hemoglobin (MCH), G) mean corpuscular hemoglobin concentration (MCHC), H) red cell distribution width - coefficient of variation (RDW-CV), I) platelet (PLT), J) platelet distribution width (PDW), K) mean platelet volume (MPV); L-O) Hepatic and renal functions examined by biochemical tests in different groups (n = 5), including L) alanine aminotransferase (ALT), M) aspartate aminotransferase (AST), N) creatinine (CRE), and O) urea (UREA). Data were expressed as mean ± SEM. One-way ANOVA analysis was used for multiple groups comparison. ns represents not significant.


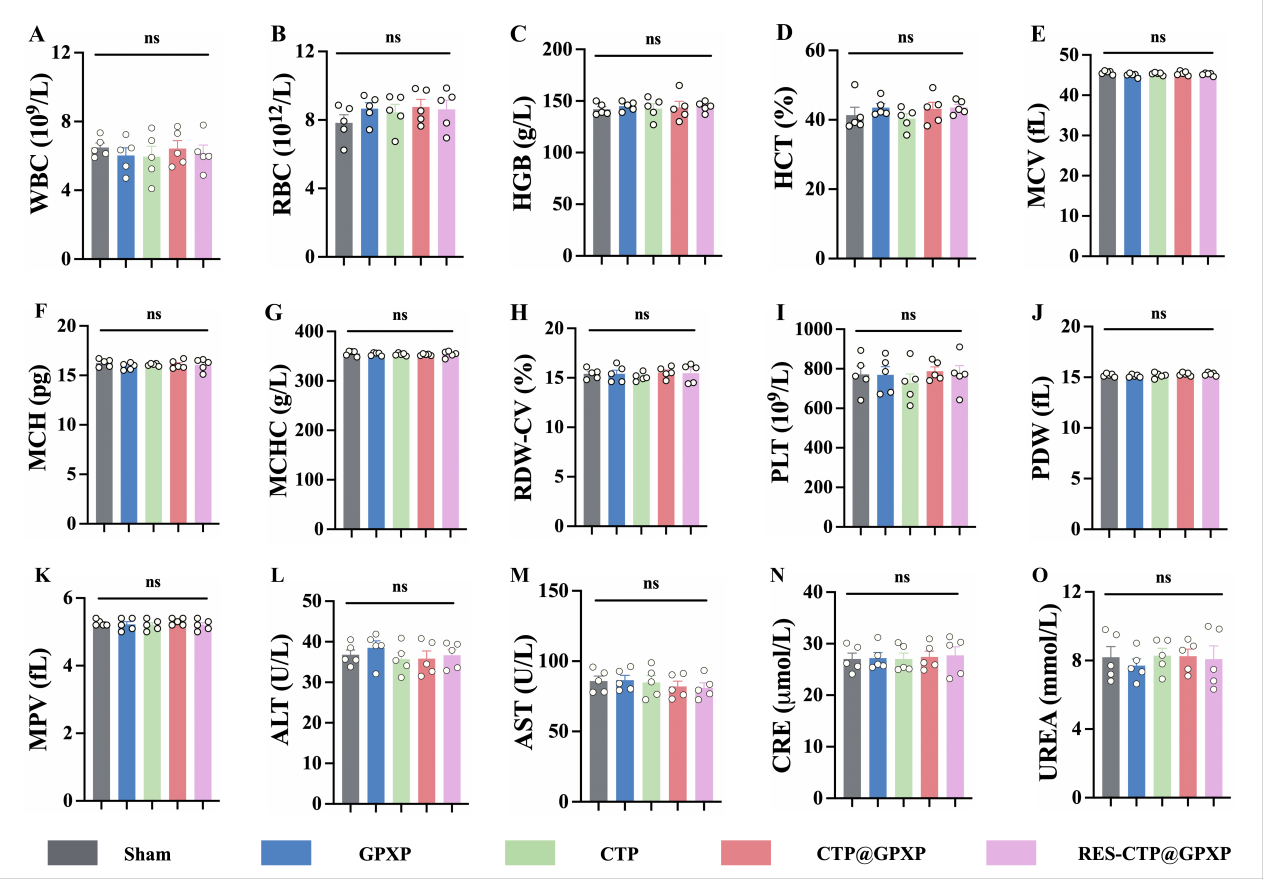


**Figure S15.** *In vivo* biosafety assessment at blood level following 28 day of drug administration in mice. (A-K) Side effects on blood cells examined by the blood routine in different groups (n = 5), including A) WBC, B) RBC, C) HGB, D) HCT, E) MCV, F) MCH, G) MCHC, H) RDW-CV, I) PLT, J) PDW, K) MPV; L-O) Hepatic and renal functions examined by biochemical tests in different groups (n = 5), including L) ALT, M) AST, N) CRE, and O) UREA. Data were expressed as mean ± SEM. One-way ANOVA analysis was used for multiple groups comparison. ns represents not significant.


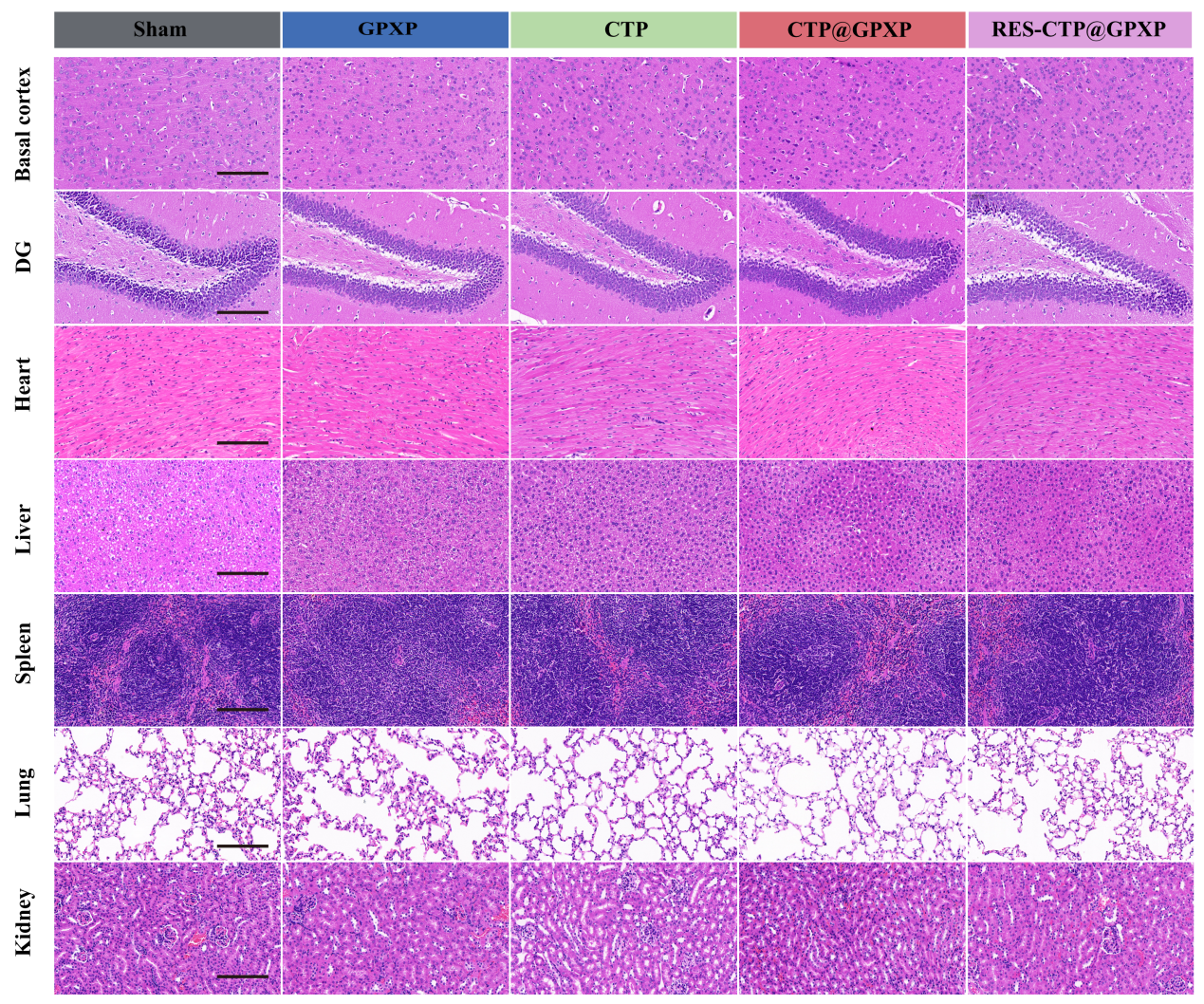


**Figure S16.** *In vivo* biosafety assessment of RES-CTP@GPXP at organ level following 7 days of drug administration in mice. Representative H&E staining of primary organs, including the brain (right basal cortex and DG region of hippocampus), heart, liver, spleen, lung, and kidney, reflecting their injury situation. Scale bars: 100 μm.

**
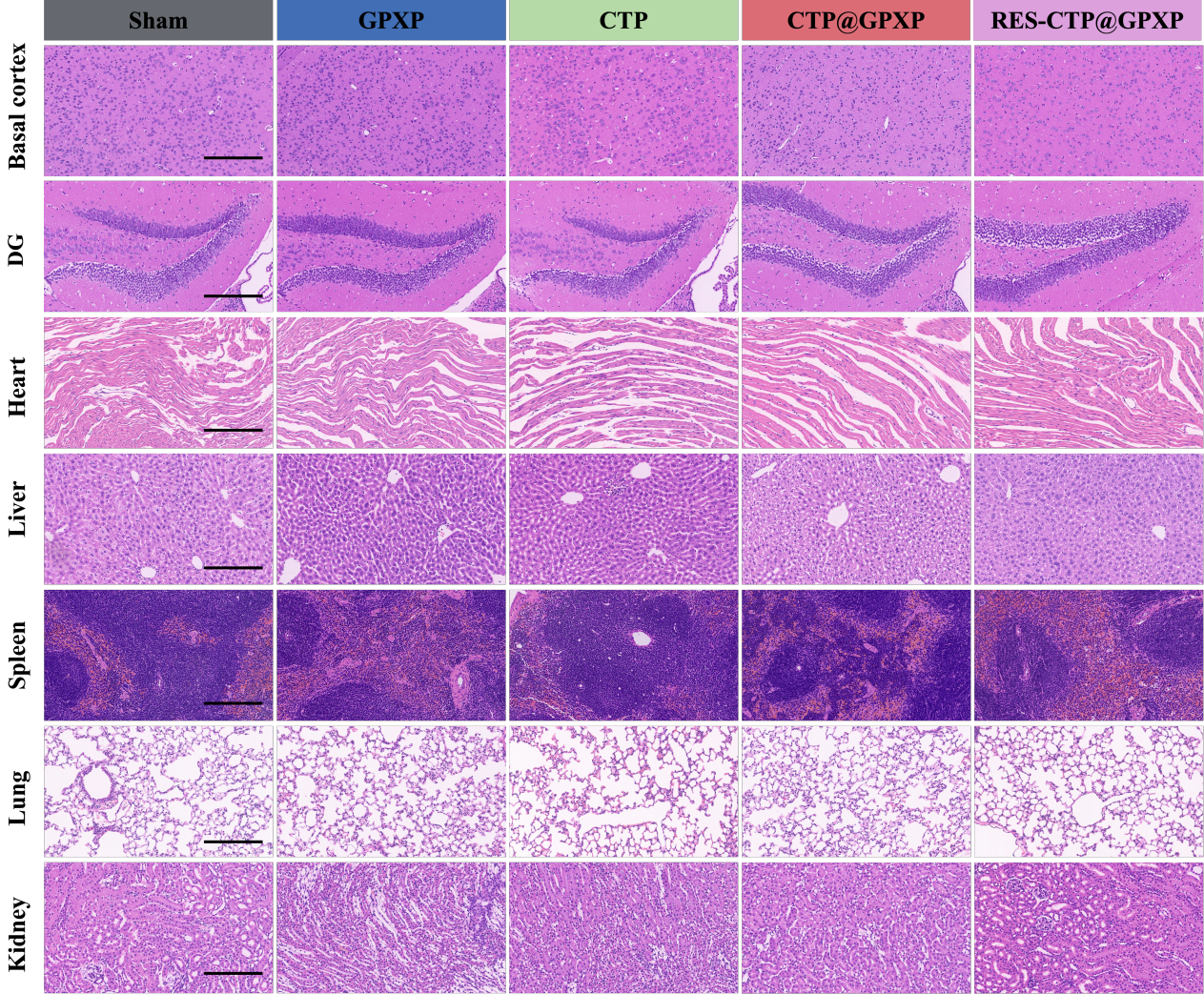
**

**Figure S17.** *In vivo* biosafety assessment of RES-CTP@GPXP at organ level following 28 days of drug administration in mice. Representative H&E staining of primary organs, including the brain (right basal cortex and DG region of hippocampus), heart, liver, spleen, lung, and kidney, reflecting their injury situation. Scale bars: 100 μm.

**
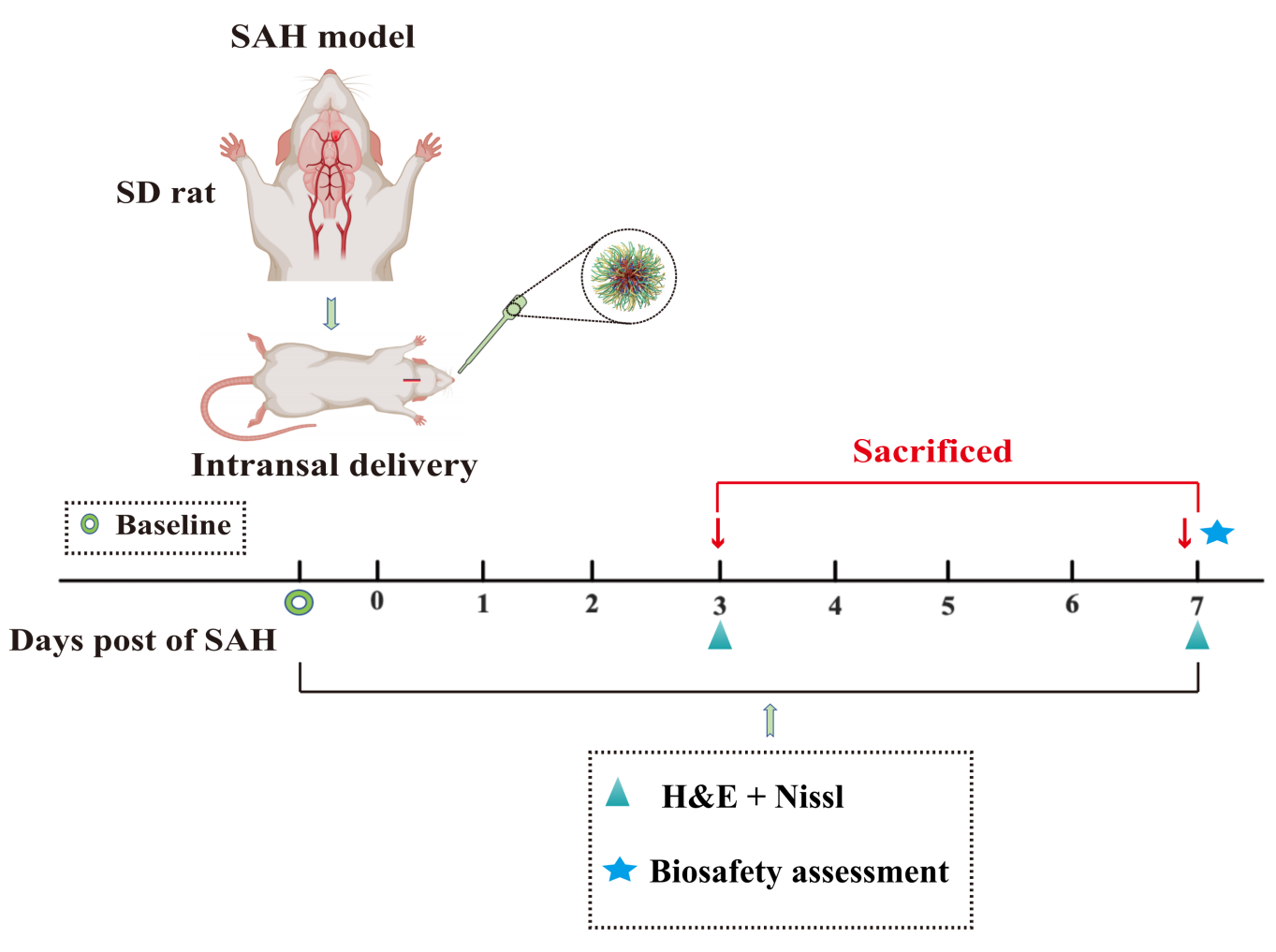
Figure S18.** The flowchart of the experiments for SD rats.


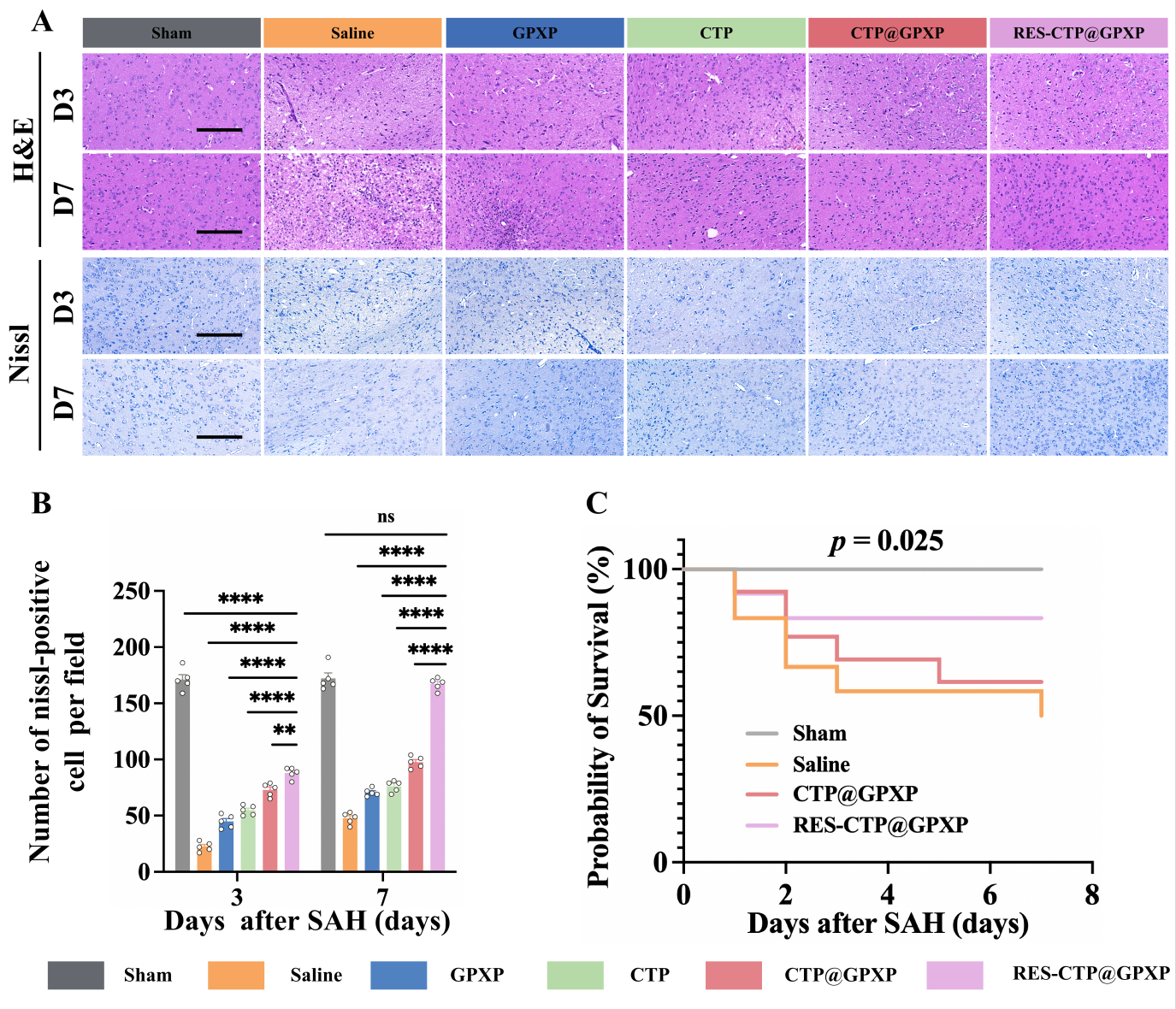


**Figure S19.** *In vivo* evaluation of improving brain injury on day 7 following SAH of SD rats. A) Representative H&E and Nissl staining at the ipsilateral basal cortex on days 3 and 7 following SAH observing changes in neuroanatomical structures. Scale bars: 100 μm. B) Quantitative analysis of the number of Nissl-positive cells by Nissl staining on days 3 and 7 following SAH (n = 5). C) Kaplan-Meier survival curve of mice upon various treatments (n = 13). Data were expressed as mean ± SEM. One-way ANOVA analysis was used for multiple groups comparison. ns represents not significant, *p* > 0.05; ^*^*p* < 0.05, ^**^*p* < 0.005, *****p* < 0.0001.


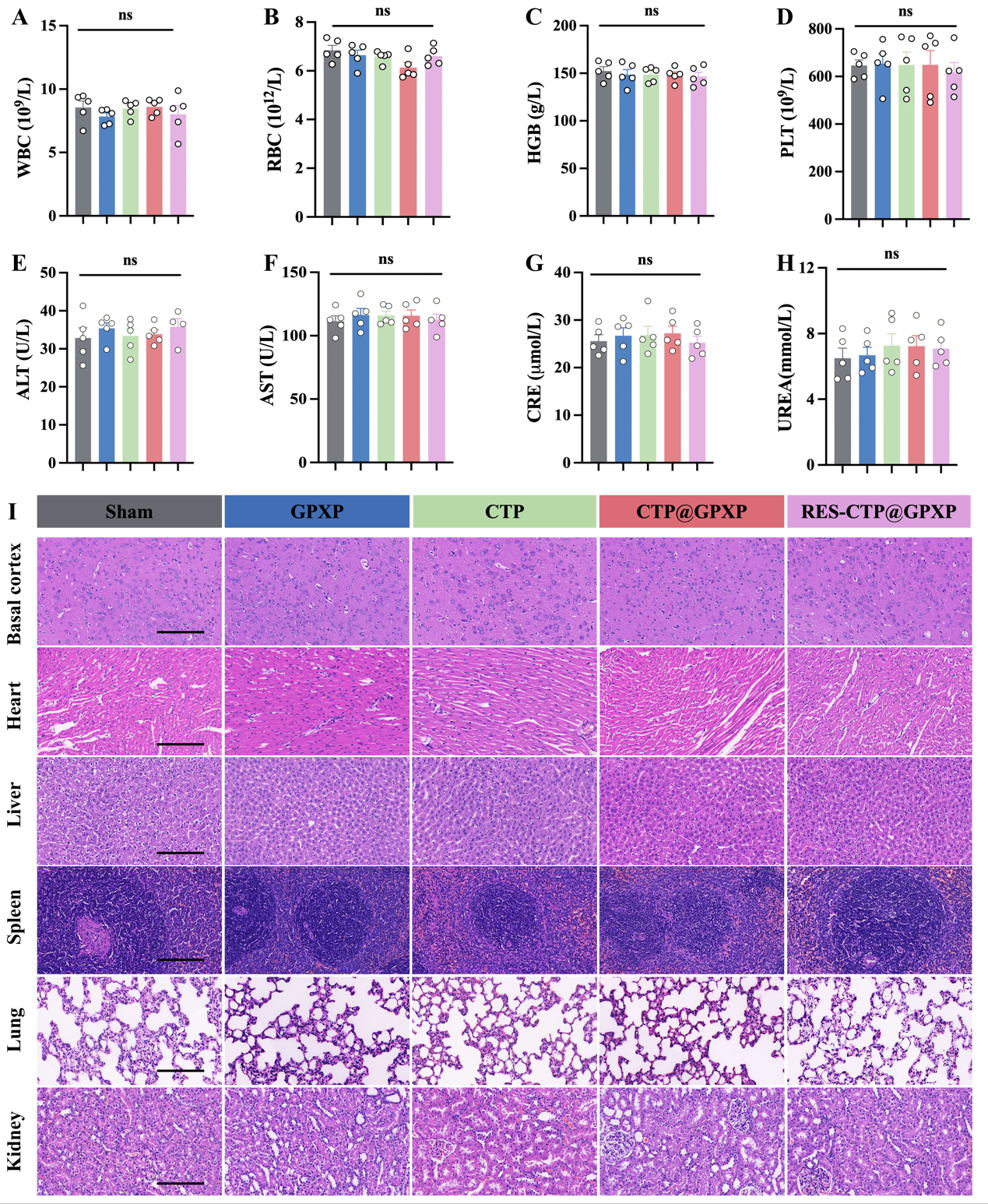


**Figure S20.** *In vivo* biosafety assessment at blood and organ levels following 7 day of drug administration for SD rats. A-D) Side effects on blood cells examined by the blood routine in different groups (n = 5), including A) WBC, B) RBC, C) HGB, and D) PLT. E-H) Liver and kidney functions examined by biochemical tests in different groups (n = 5), including E) ALT, F) AST, G) CRE, and H) UREA. I) Representative H&E staining of primary organs, including the brain (right basal cortex), heart, liver, spleen, lung, and kidney, reflecting their injury situation. Scale bars: 100 μm. Data were exhibited as mean ± SEM. One-way ANOVA analysis was used for multiple comparison. ns represents not significant.


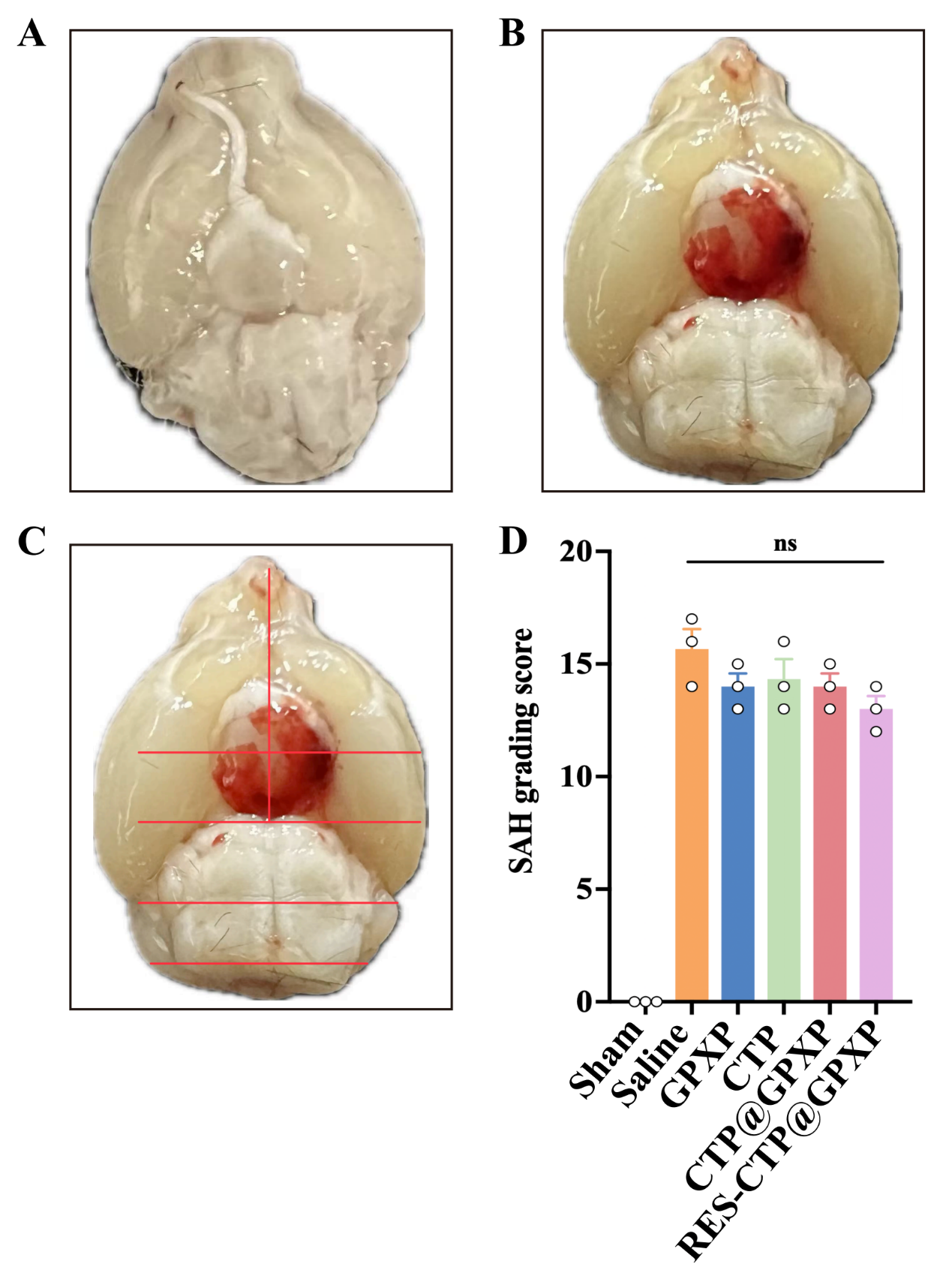


**Figure S21.** Anatomical evaluation and SAH grade scores. A, B) Representative anatomical images of sham and SAH mice. C) Schematic of SAH grading method, showing the division of the basal cistern into 6 segments. D) SAH grading scores in each group (n = 3). ns represents not significant.

**Tables**

**
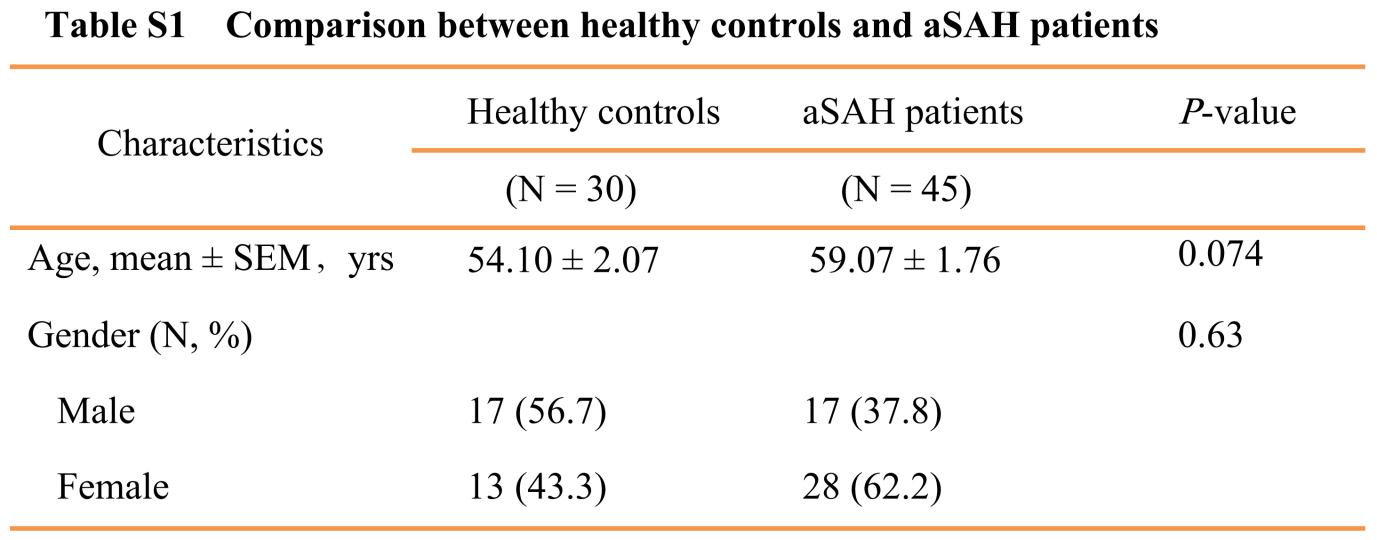
Table S1** Comparison between healthy controls and aSAH patients.


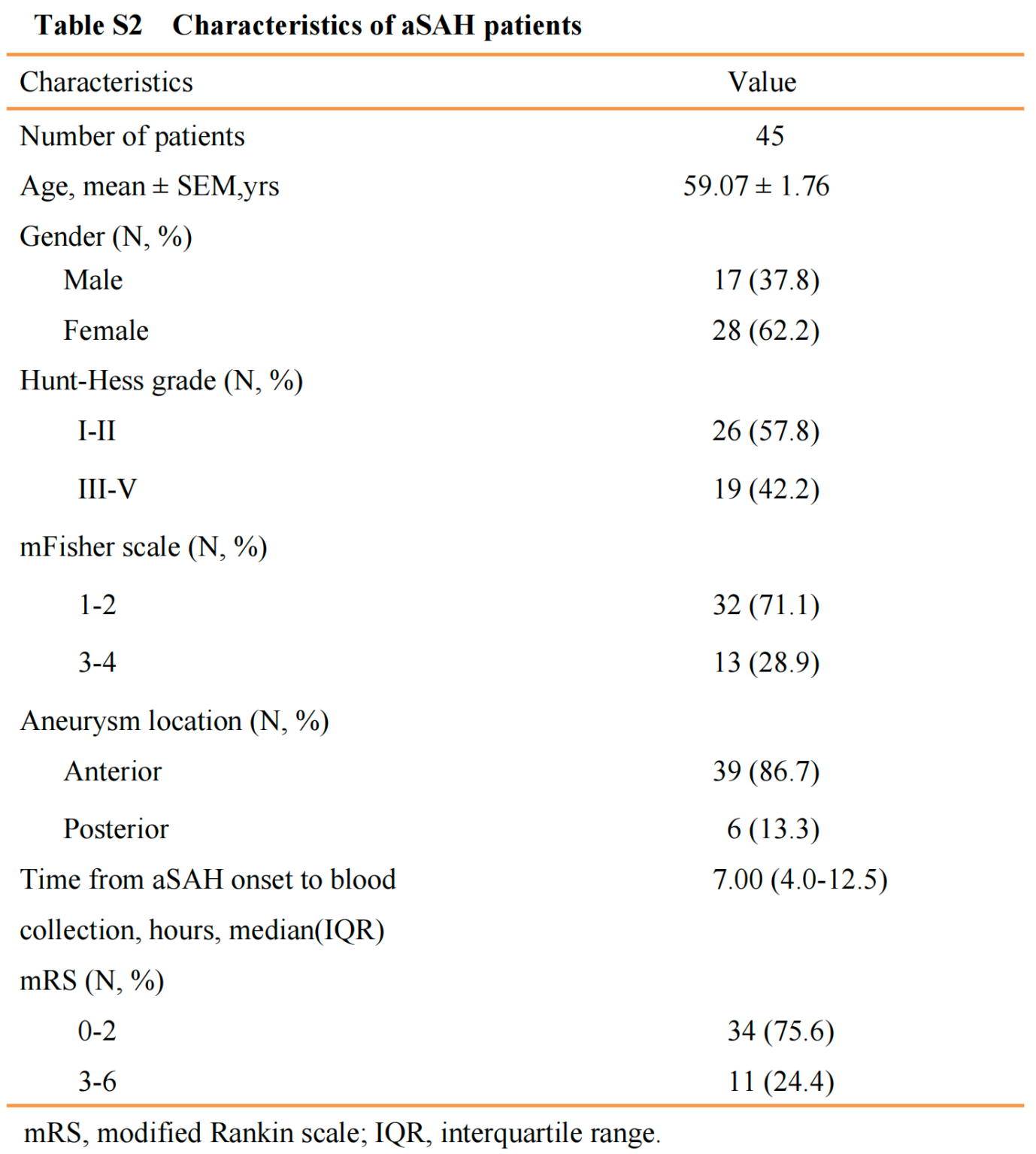
**Table S2** Characteristics of aSAH patients.


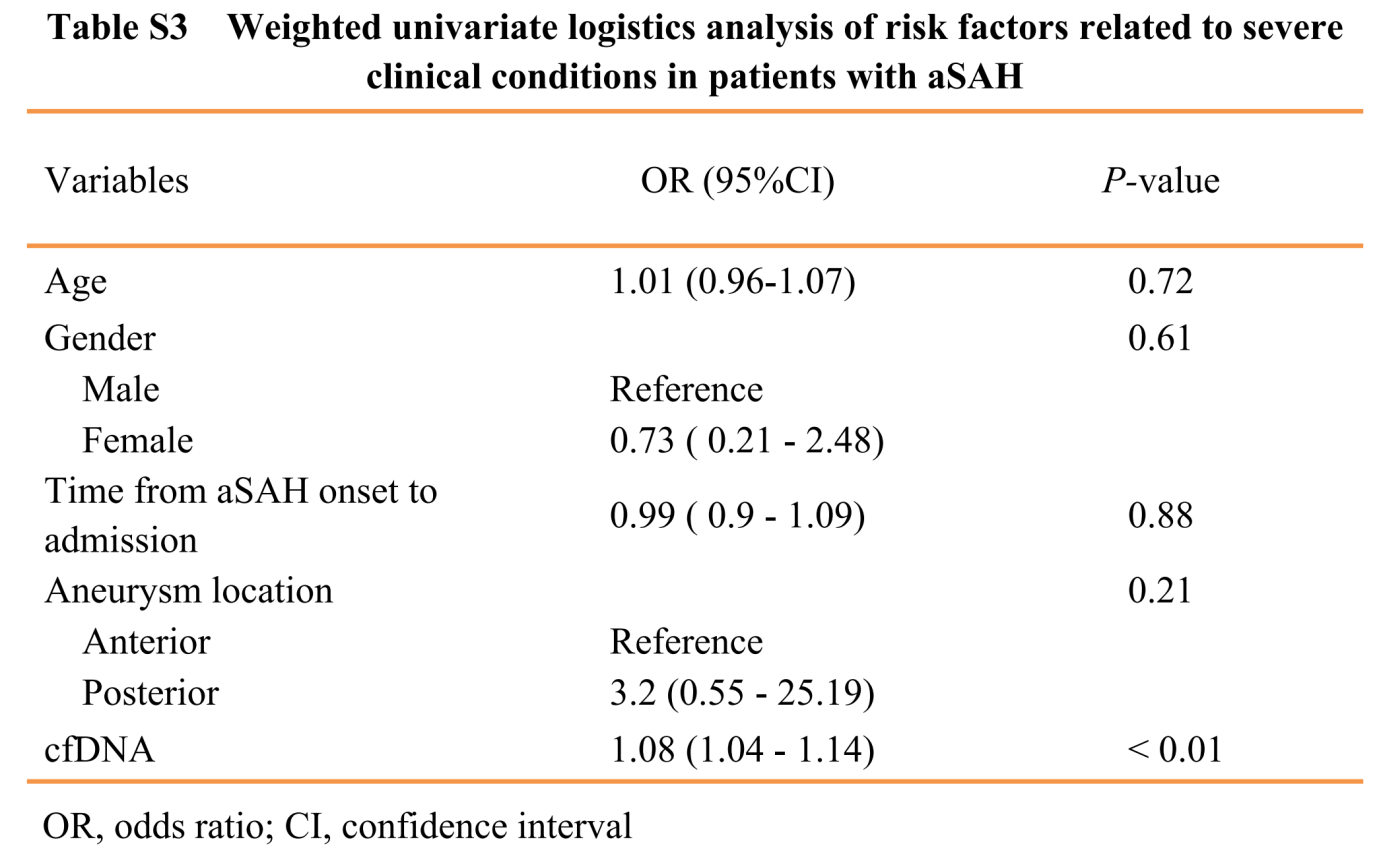
**Table S3** Weighted univariate logistics analysis of risk factors related to severe clinical conditions in patients with aSAH.


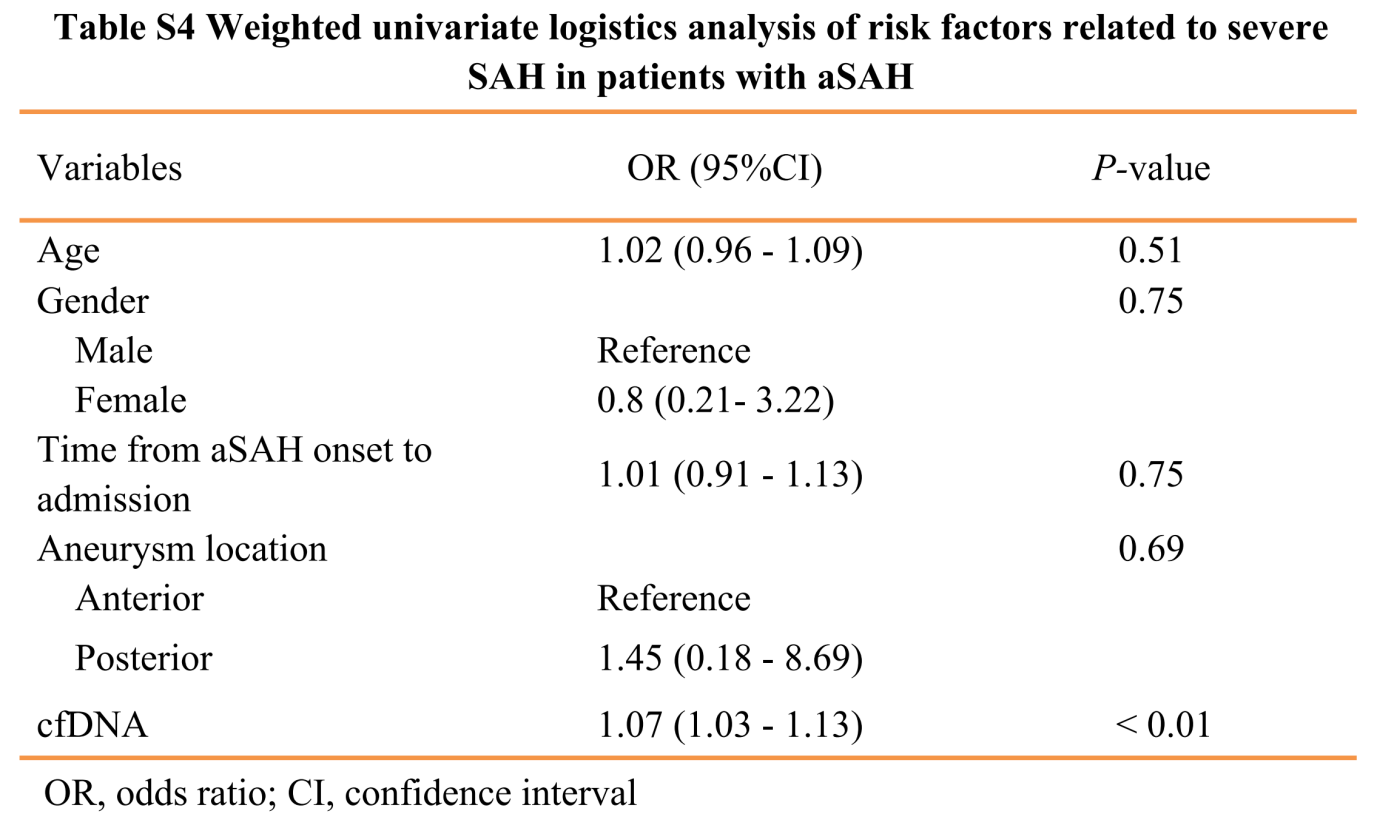


**Table S4** Weighted univariate logistics analysis of risk factors related to severe SAH in patients with aSAH.


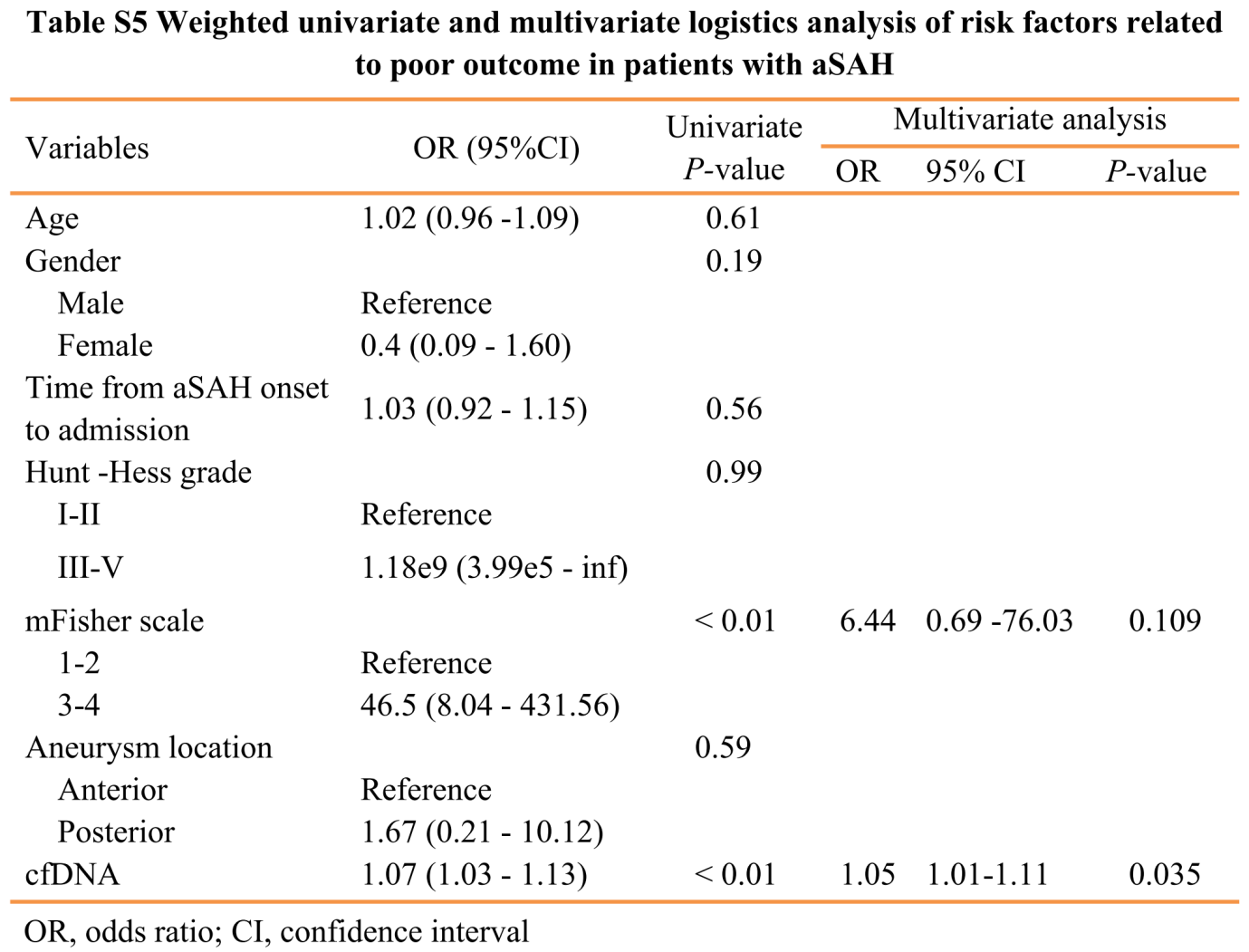


**Table S5** Weighted univariate and multivariate logistics analysis of risk factors related to poor outcome in patients with aSAH.

**References**

[1] D. Feng, J. Zhou, H. Liu, X. Wu, F. Li, J. Zhao, Y. Zhang, L. Wang, M. Chao, Q. Wang, H. Qin, S. Ge, Q. Liu, J. Zhang, Y. Qu, Astrocytic NDRG2-PPM1A interaction exacerbates blood-brain barrier disruption after subarachnoid hemorrhage. *Sci. Adv.* **2022**, *8*, eabq2423.

[2] X. Hu, J. Yan, L. Huang, C. Araujo, J. Peng, L. Gao, S. Liu, J. Tang, G. Zuo, J. H. Zhang, INT-777 attenuates NLRP3-ASC inflammasome-mediated neuroinflammation via TGR5/cAMP/PKA signaling pathway after subarachnoid hemorrhage in rats. *Brain Behav. Immun.* **2021**, *91*, 587.

[3] J. F. Alexander, A. V. Seua, L. D. Arroyo, P. R. Ray, A. Wangzhou, L. Heibeta-Luckemann, M. Schedlowski, T. J. Price, A. Kavelaars, C. J. Heijnen, Nasal administration of mitochondria reverses chemotherapy-induced cognitive deficits. Theranostics 2021, 11, 3109.

[4] H. Kanamaru, S. Zhu, S. Dong, Y. Takemoto, L. Huang, P. Sherchan, H. Suzuki, J. Tang, J. H. Zhang, UDP-Glucose/P2Y14 Receptor Signaling Exacerbates Neuronal Apoptosis After Subarachnoid Hemorrhage in Rats. *Stroke* **2024**, *55*, 1381.

[5] T. Okada, B. Enkhjargal, Z. D. Travis, U. Ocak, J. Tang, H. Suzuki, J. H. Zhang, FGF-2 Attenuates Neuronal Apoptosis via FGFR3/PI3k/Akt Signaling Pathway After Subarachnoid Hemorrhage. *Mol. Neurobiol.* **2019**, *56*, 8203.
